# Improved DNA extraction on bamboo paper and cotton is tightly correlated with their crystallinity and hygroscopicity

**DOI:** 10.1371/journal.pone.0277138

**Published:** 2022-11-07

**Authors:** Xiaojun Ye, Bo Lei

**Affiliations:** 1 Division of Science and Technology, Beijing Normal University - Hong Kong Baptist University United International College (UIC), Zhuhai, Guangdong, China; 2 Department of Chemistry, Faculty of Science, Hong Kong Baptist University (HKBU), Hong Kong, China; USDA-ARS Southern Regional Research Center, UNITED STATES

## Abstract

DNA extraction, a vital pre-requisite for most biological studies, continues to be studied extensively. According to some studies, DNA shows a certain degree of absorbability on filter paper made of plant fiber-based adsorbent material. However, the principle underlying such specific adsorption as well as plant species associated with plant fiber-based adsorbents and optimized extraction conditions have not yet been studied. This study demonstrates the tight correlation between crystallinity and hygroscopicity in plant fiber-based adsorbents used for DNA extraction and proposes the concept of DNA adsorption on plant fiber-based adsorbents, for the first time. We also explored optimal extracting and eluting conditions and developed a novel plant fiber-based DNA extraction method that was quadruple times more powerful than current approaches. Starting with the screening of various types of earthed plant fiber-based adsorbents, we went on to mine new plant fiber-based adsorbents, bamboo paper and degreased cotton, and succeeded in increasing their efficiency of DNA extraction to 4.2 times than that of current approaches. We found a very strong correlation between the crystallinity and hygroscopicity of plant fiber-based adsorbents which showed efficiency for DNA extraction, and thus propose a principle that potentially governs such specific adsorption processes, in the hope that this information may guide related multidisciplinary research studies in the future. Nanodrop, electrophoresis and PCR were selected to demonstrate the quantity, quality, integrity and utility of the extracted DNA. Furthermore, crystallinity, hygroscopicity, pore size distribution and composition of plant fiber-based adsorbents were studied to explore their correlation in an attempt to understand the principle underlying this particular type of adsorption. The findings of this study may be further extended to the extraction of other types of nucleic acids with similar biochemical properties.

## 1. Introduction

### 1.1 The crucial significance

Nucleic acid extraction is an essential prerequisite for most molecular analyses, including nucleic acid amplification, DNA fragment amalgamation, vector construction and high-throughput sequencing [1–3]. With respect to life sciences, nucleic acid amplification may be used for plant pathogen detection, food detection and disease diagnoses [4–7]. Following the development of biotechnology, gene detection has gradually evolved into a new technology showing potential for disease and health analysis as well as for traditional physical examinations [8–11]. The accuracy and reliability of the downstream outcome of an analysis depends on upstream extraction and purification of nucleic acids [12]. Although technologies associated with downstream biological detection are on the rise [13], corresponding technological input to upstream nucleic acid extraction technology appears to be inadequate. Currently, increasing the capacity for nucleic acid testing is tantamount to an increase in the capacity to cope with the number of samples tested, particularly in relation to pandemic situations such as the recent COVID-19 outbreak. Improvement in extraction efficiency becomes equally concerning because only effective nucleic acid extraction can guarantee the accuracy and reliability of downstream decision-making. The high percentage of false negative results associated with COVID-19 testing, indicates the inadequacy of current nucleic acid extraction techniques [14, 15]. Improving nucleic acid extraction essentially involves enhancing the quantity of extracted nucleic acid while maintaining its purity. This indicates that continuous optimization and improvement of nucleic acid extraction technology is of great significance [3, 16, 17]. As the biochemical characters of nucleic acids are similar [3], DNA extraction was selected as the model in this study.

### 1.2 Current approaches for DNA extraction

Based on differences between the media used, DNA extraction technique is divisible into liquid (traditional) and solid phase extraction. Liquid phase DNA extraction involves the use of toxic organic solvents (i.e., phenol and chloroform) as the core extracting media. These solvents are considered to be environmentally unfriendly in addition to being associated with cumbersome procedures, health risks to humans, and incomplete phase separation [2]. Therefore, researchers tend to use commercial kits intended for solid phase extraction. Solid phase extraction utilizes interaction between a solid adsorbent (usually silica-based) and DNA to achieve extraction and purification [3, 18–20]. Compared with liquid phase extraction techniques, solid phase extraction techniques are highly efficient and do not involve the disadvantages mentioned above. Thus, solid phase extraction has currently come to be the mainstream DNA extraction.

“Centrifuge column (CC)” and “magnetic bead (MB)” methods are the two main commercial solid phase DNA extraction techniques, where CC kits have been widely used [21–24]. The CC method, usually referred to as the silica-based DNA extraction method, was first established in 1979 [25]. Current theory maintains that the adsorption between DNA and silica is mediated by a salt bridge under low pH and a high salt condition [2, 3, 26]. The silanol groups in silica and in DNA extracted from samples are generally negatively charged, leading to mutual repulsion. An appropriate concentration of cations neutralizes the negative charges, thereby mediating adsorption between these two [27] (S1 Fig). When using the CC kit, the adsorbent membrane in the CC (usually silica membrane) specifically adsorbs DNA from the sample lysates (e.g., the cell lysates) following which the remaining cell lysate is removed via centrifugal force, due to a lack of mutual attraction between the silica membrane and the cell lysate remaining after adsorbed DNA has been finally eluted by the eluant [2, 13, 27]. The MB method is also a feasible solid phase extraction technique. The main principle of the MB method is similar to that of the CC method, because target DNA is specifically adsorbed on a silica-based membrane coated on MBs (S1 Fig). Thus, MBs adsorb DNA specifically while undergoing serial cleaning to remove impurities [27–29] thereby achieving DNA extraction after elution. Current technology uses magnetic nanoparticles usually coated with a silicon dioxide (SiO_2_) shell layer which exhibits good hydrophilicity, non-toxicity and protectiveness [1]. Non-recyclability and tendency to aggregate are factors that need to be considered during MB extraction.

### 1.3 Obstacles brought by the current approaches

Due to ease of use, commercial kits have become the first choice for nucleic acid extraction. Nucleic acid extraction kits, represented by DNA extraction kits, only provide instructions and do not divulge any information regarding the buffers and adsorbents involved. While commercial DNA extraction kits provide convenience and ease of use to scientific researchers, some senior researchers contend that commercial DNA extraction kits are stifling creativity and blinding researchers, thereby hindering the advancement of DNA extraction technology and preventing innovation [30, 31]. Focus on DNA extraction itself has been stymied to keep pace with the progress of downstream analysis. These may be the main reasons for the objections raised by senior scientists. Thus, the necessity for continuing innovation in the area of DNA extraction cannot be overemphasized. While enjoying the advantages conferred by DNA extraction kits, researchers should also confront issues associated with these kits and respond appropriately. Due to confidentiality issues pertaining to the release of technical information relevant to DNA extraction kits, research on innovative DNA extraction is faced with certain challenges linked to information asymmetry. The efficacy of innovative DNA extraction techniques needs to be compared with currently used approaches. This study implemented an innovative DNA extraction scheme using new plant fiber-based adsorbents (PF-BAs). Here, we have described, as specifically as possible, detailed information regarding the adsorbents and the system of extraction buffers used to achieve DNA extraction with considerable improvements in quantity and quality.

### 1.4 Progress of current research for plant fiber-based adsorbents (PF-BAs) for DNA extraction

PF-BAs may be more preferable compared with cellulose-based adsorbents (from previous studies), because a fibrous structure is a prerequisite for adsorption as demonstrated by the results of this study (Part 3.3). Recent studies of DNA adsorption on PF-BAs based on commercial filter papers are limited. Recent progress in the application of commercial filter papers (not limited to PF-BAs) may be summarized as follows: In 2020, *Mason and Botella* designed a 50 mm-long and 2 mm-wide DNA extracting paper dipstick, made of Whatman^™^ Grade 1 filter paper and offered a protocol for using this dipstick, claiming that it could be applied on a variety of samples, such as cells, tissues and plants, that can be directly subjected to PCR detection [32]. This concept originated in 2017, when they first produced the dipstick, claiming that Whatman^™^ Grade 1 filter paper could adsorb DNA extracted from plants. Later, they expanded the use of Whatman^™^ Grade 1 dipsticks to a variety of samples. The protocol provided was specific for samples that could be directly subjected to PCR, and no direct DNA-related quantitative or qualitative data was provided [33]. In 2018, Shi *et al*., selected and implanted the Whatman^™^ Grade 3 and GF/F filter into recharged commercial and homemade spin columns. QIAGEN^®^ DNeasy Plant mini kit and homemade buffers were applied in recharged and homemade QIAGEN^®^ columns. The yield of DNA extracted from tomato leaves using homemade buffer was higher than that was extracted using QIAGEN^®^ ones. However, the purity of most DNA extracted using homemade buffer was far from desirable [34]. In 2014, Qiu and Mauk installed Whatman^™^ FTA membrane as the DNA adsorbent on their specific microfluid chip with a single chamber and claimed that DNA extraction as well as the related PCR detection could be accomplished simultaneously. The DNA extracting capacities of two operating modes (filtration and diffusion modes) and two different time flows (single and triple time) were compared, using *Bacillus cereus* as a model sample; no data pertaining to DNA qualification and quantification were provided [35]. Gan *et al*., (2014) compared the DNA extracting capacity of Whatman^™^ Grade 1, 2, 3, 5, 602h and Fusion 5 filter papers using the designed microfluid chip and microdevice, and reported that the Fusion 5 filter showed the strongest capacity for extracting DNA among the tested filters. The DNA extracted on-chip via the Fusion 5 filter, using human whole blood as the sample model, was quantitatively superior to that extracted via the QIAamp DNA Micro Kit. Next, the applicability of the Fusion 5 filter on-chip method was tested on other samples, such as saliva and cigarette butts; no related purity data were provided [36].

### 1.5. Research gaps and purpose of the current study

The above-mentioned studies mainly verified the feasibility of using commercial filter paper to extract DNA by directly implanting filters into a DNA extraction column, a microfluid chip or a specific device. The applications of PF-BAs were limited to commercial filters and research studies that were conducted on PF-BAs were neither comprehensive nor in-depth. Firstly, the research on PF-BAs was limited to the application stage only. Thus, the mechanisms underlying the adsorption of DNA on PF-BAs remained unclear [33]. Secondly, the coverage of PF-BAs was insufficient in that mostly Whatman^™^ Grade 1 was utilized, whereas Grade 3 had already been reported as showing efficiency comparable to that of commercial extraction kits. Thirdly, the actual DNA extraction capacity of the selected commercial filters was ignored because most studies directly transferred the extracted solution to the detection stage for rapid results. Fourthly, the filters were commercial products and thus core information (i.e., composition) as well as the methods used to manufacture these filters remained trade secrets. Implanting them on DNA extraction kits could lead to better extraction efficiency than that of the existing kits, but this was tantamount to conducting a “double-blind” experiment which led to a desirable result, without being aware of the ingredients that were being used. From the perspective of innovation, it is scarcely possible to conduct further exploration without knowing the details of experimental materials. Fifthly, quantity and quality are comprehensive indexes that can be used to evaluate DNA extraction techniques. On one hand, the quantity of extracted DNA indicates the efficiency of the extraction technique, where the larger the extracted quantity the more the accuracy of downstream detection results. Especially, the extraction efficacy of DNA is particularly important in cases where only small amounts of the target DNA sequences are present in the sample. On the other hand, quality indicates the purity of extracted DNA that could be utilized in downstream experiments when it meets the criteria. If the extracted sample does not reach a specific purity, it would affect the accuracy of results as well as the reliability of downstream experiments, thereby narrowing the range of utilization [30].

The ratios of OD260/OD280 and OD260/OD230 together can accurately reflect the purity of extracted DNA. Currently, most studies only provide the OD260/OD280 ratio of extracted DNA, whereas the OD260/OD230 ratio tends to be ignored. The OD260/OD280 ratio alone is insufficient to estimate the purity of extracted DNA, because DNA shows strong absorption at OD260, as a result of which only high protein concentration can be detected via a change in the OD260/OD280 ratio [37]. OD260/OD230 may also be considered as key purity data, especially for downstream analyses which require highly pure nucleic acids [38]. Protein residues result in a significant increase in OD230 which, in turn, significantly affects the OD260/OD230 ratio [39]. More specifically, an OD260/OD280 ratio larger than 2.0 indicates contamination by RNA while a ratio less than 1.7 indicates the existence of proteins and phenolic compounds; an OD260/OD230 less than 2.0 indicates the presence of residual polysaccharides, salts or other organic solvents [40, 41]. Therefore, extracted DNA of high-quality is expected to have an OD260/OD280 ratio within 1.7 to 2.0 and an OD260/OD280 ratio over 2.0 [30]. Bypassing purity makes any discussion of extraction incomplete. Many downstream experiments have indicated that qualified purities and highest possible quantities of extraction may ensure the universality of extracted DNA.

Based on the criteria discussed above, this study widened its focus on PF-BAs by screening a variety of PF-BAs to provide a more comprehensive picture of the DNA extracting capacity of PF-BAs (Part 3.2). This would expectedly deepen our understanding of the characteristics of PF-BAs (Part 3.3), thereby achieving a close to ideal outcome of DNA extraction (Part 3.4), revealing strong correlation between it and extraction efficiency (Part 3.5) and clarifying the principle underlying specific adsorptions (S9 Fig). This study attempted to take the initiative by establishing and optimizing DNA extraction based on PF-BAs to prove its superiority over existing DNA extracting methods, with reference to quantity while retaining purity related criteria (S3 Fig). We aimed to elucidate the composition of each buffer, and expose the principles underlying the adsorption of DNA on PF-BAs, by investigating crystallinity, hygroscopicity, zeta potential, pore size and composition ratios. Mastering experimental materials, procedures and principles may hopefully reveal more possibilities for present and future studies, thereby enhancing the scope of innovative research in the future. The applications of PF-BAs has been limited to commercial filters, and studies on PF-BAs have not been sufficiently comprehensive.

## 2. Materials and methods

### 2.1. Assembling the novel extraction spin column sets

Creation of special spin columns was based on purchasing empty nucleic acid extraction column sets, including internal columns (with supporting parts at the bottom), external columns, and fixing collars (HAOQI). The method used to construct each extraction spin column set was as follows: one layer of a qualitative filter paper (SHUANGQUAN^®^) disc was loaded onto the supporting part of the internal column which performed the supporting role, following which certain amounts of selected adsorbents (Table 1) were loaded onto the supporting filter paper disc; using the end of a 200 μL tip (KIRGEN^®^), the fixing collar was pushed tightly onto the loaded adsorbent to hold the stuffing; the assembled internal column was then loaded into the external column forming an extraction spin column set. The assembled extraction spin column sets were collected in sterilized boxes, sealed in sterilized bags, autoclaved at 121°C for 30 min (YAMATO SQ810C), dried in a drying oven (MEMMERT) at 60°C for 30 min, and kept in a benchtop biosafety cabinet (BSC); (AIRTECH) prior to use. All loaded discs were punched using a customized 7 mm hole puncher (HONGXUAN); (S2 Fig). Each assembled special extraction spin column was prepared in triplicate (n = 3).

**Table 1:** Details of the selected adsorbents.

Adsorbents	Pieces/Column	Price*($/m^2^)	Supporting disc^1^	Brands^2^
**Grade 1 filter paper**	1 to 20 disc	25	No	Whatman^™^
**Grade 3 filter paper**	1 to 10 disc	29	No	Whatman^™^
**GF/F microfiber filter**	1 to 15 disc	1620	Yes	Whatman^™^
**Bamboo paper (BP)**	1 to 30 disc	0.065	Yes	PsanMuve
**Wood paper (WP)**	30 disc	0.12	Yes	QINGFENG^®^
**Straw paper (SP)**	30 disc	0.18	Yes	QUANLIN^®^
**Degreased cotton (DC)**	0.084 g (±0.002)	0.0057/g	No	HLK

^1^The use of supporting paper depended on the texture of the adsorbent.

^2^Related prices were provided by the specialized reagent purchasing company.

### 2.2. DNA extraction via the novel spin column set

Exploration of DNA extraction started with the implanting of selected PF-BAs into the special spin column set, whereupon current protocols were applied to determine whether it is worthwhile to use PF-BAs for further optimization (Part 2.3). In this case, the commercial DNA extraction kits, DNeasy Blood & Tissue Kit (QIAGEN^®^), TIANamp Genomic DNA Kit (TIANGEN^®^), Blood Genomic DNA Extraction Kit (NANOEAST^®^), Tissue/cell Genomic DNA Extraction Kit (AIDLAB^®^) and Mammalian Genomic DNA Extraction Kit (BEYOTIME^®^), covering liquid and solid phases, were selected to determine the current status of DNA extraction and provide objects for comparison. Related operations were performed according to provided instructions (S4 Fig). Each procedure was performed in triplicate (n = 3). Next, a related innovative protocol was developed in response to targeted adsorbents. The innovative protocol for DNA extraction was developed after repeatedly practicing with TIANGEN^®^, QIAGEN^®^ and NANOEAST^®^ and analyzing related published protocols [32, 33]. Further, the new protocol was optimized via an implemental process to achieve extraction effect as ideal as possible (Part 2.3).

### 2.3. Innovative approach for PF-BA based DNA extraction

The basic recipes of lysis buffer C [42], lysis buffer N [32] and elution buffer Q [43] are shown (Table 2). The pH values of the related lysis buffers were adjusted from pH 4 to 10 using hydrochloric acid (HCl) (36.0~38.0%) (GUANGSHI) and NaOH powder (YONG DA) via real-time monitoring using a pH meter (SANXIN MP521). Prior to each pH adjustment of the related buffers, the pH meter was corrected using a pH correcting buffer solution at pH values of 4.00, 7.00 and 10.00, at room temperature (GUANGJIAN). All solutions were prepared using sterilized enzyme-free water (SOLARBOI^®^), filtered using a 0.2 μm sterilized syringe filter (PALL^®^), prepared, stored and used in a benchtop BSC (AIRTECH).

**Table 2:** The buffers and adsorbents.

Content	Recipe
**Lysis buffer C**	100 mM Tris-Cl, 50 mM EDTA (YUANYE^®^), 1% SDS [42], pH 4 to 10 (±0.01)
**Lysis buffer N**	800 mM guanidine hydrochloride, 50 mM Tris, 0.5% (v/v) Triton X-100, 1% (v/v) Tween 20 [32], pH 4 to 10 (±0.01)
**Adsorbents (in the internal column)**	WP (QINGFENG^®^), SP (QUANLIN^®^), BP (PsanMuve) and DC (HLK), c.powder^1^ (ALADDIN^®^)
**Washing buffer E**	70% ethanol
**Elution buffer Q**	10 mM Tris-Cl, 0.5 mM EDTA, pH 6 to 10 (±0.01)

^1^c. powder refers to the cellulose powder (grain size: 90 μm)

### 2.4 Protocol for the new approach

(i) First, 2.0*10^6^ cell were centrifuged for 2 min at 900 x *g* and the supernatant discarded. Next, 200 μL PBS (SOLARBIO^®^) was added and mixed properly; (ii) then, 20 μL proteinase k (SOLARBIO^®^) was added followed by 200 μl Lysis buffer, and mixed gently while pipetting to yield a homogeneous solution; (iii) 200 μl absolute ethanol (DAMAO^®^) was added to the lysate and mixed gently while pipetting to yield a homogeneous solution (note- the sample and the ethanol must be mixed thoroughly to yield a homogeneous solution); (iv) pipet the solution from step (iii) into the internal (adsorbent) column, and wait for approximately 1 min to let the adsorbent adsorb the solution; (v) place the internal column in the external column, centrifuge at 900 x *g* for 2 min, and discard the effluent; (vi) Add 600 μL of 70% ethanol, centrifuge at 900 x *g* for 2 min and discard the effluent; (vii) repeat step (vi); (viii) place the internal column in a 1.5 mL or 2 mL sterilized centrifuge tube, add 200 μL elution buffer into the internal column, and wait for approximately 1 min to let the adsorbent absorb the buffer; (ix) Centrifuge at 900 x *g* for 2 min and collect the effluent. The effluent is the extracted DNA solution.

### 2.5. Scanning electronic microscopy

Images of selected PF-BAs were captured and magnified to 1000x using a scanning electron microscope (ZESIS EVO 18, Germany) with extra high tension (EHT) at 10.00 kV and a working distance (WD) of 9.5 mm.

### 2.6. Crystallinity analysis

XRD equipped (Rigaku Ultima IV, Japan) with Cu Kα radiation in the 2θ range 3–80° with a step size of 0.02°, was used under the operational conditions of 40 mA, 40 kV and scanning speed 10°/min. The amorphous index (AmorI) can be calculated from the height ratio between intensity of crystalline peak and total intensity of non-crystalline peak subtracted by 1 using the formula below.

$$AmorI=1-\frac{I_{002}-I_{am}}{I_{002}}\times 100\%$$

where *AmorI* indicated the amorphous index; *I*_*am*_ is the intensity of amorphous region; and *I*_*002*_ is the intensity of crystal plane.

### 2.7. Hygroscopicity measurements

The contact angle was determined using a contact angle goniometer (OCA Dataphysics, Germany). The sessile drop technique was applied under ambient conditions (room temperature) for flat sheet membranes. Distilled (DI) water (1 μL) was dropped on the surface of selected PF-BAs and images of droplets present on the surface were captured immediately.

### 2.8. Measurement of pore size distribution

Nitrogen adsorption-desorption isotherms were determined using an SA 3100 (BECKMAN COULTER). PF-BAs were degassed at 60°C under vacuum conditions. The Barret, Joyner and Halenda (BJH) model method was used to calculate the pore size distribution of samples.

### 2.9. Determination of cellulose, hemicellulose and lignin

The hydrolysate was obtained from the National Renewable Energy Laboratory (NREL) [44]. Hemicellulose and cellulose were determined via the ultraviolet spectroscopy of orcinol and anthrone [45] lignin was determined using ultraviolet spectrophotometry and the weighing method [46].

#### Water-ethanol extraction

A 2 to 3 g sample was weighed in a filter paper cylinder that had been weighed, the filter paper cylinder was placed in the Soxhlet extractor, 200 mL water was added, refluxed for one hour, air dried, treated with another 200 mL of anhydrous ethanol, refluxed for one hour, and air dried.

#### Two-step acid hydrolysis

First, 300 mg (*wef*) of the above treated sample was weighed in a reagent bottle, 8 mL of 72% H_2_SO_4_ was added, shaken well, and placed in a water bath at 30°C for 1 h. Then 224 mL distilled water was added and the above samples was placed in an autoclave at 121°C for 1 h. After the hydrolysate was cooled to room temperature, the filtrate was placed in a glass sand core crucible (G3), following which approximately 50 mL of filtrate was collected and stored at 0 to 4°C. Lignin was determined within 6 h, and cellulose and hemicellulose within 24 h.

#### Lignin: Acid-soluble lignin

The absorbance value of the acid hydrolysate was measured at 320 nm with a UV-visible spectrophotometer, using distilled water as the blank. The sample was diluted with distilled water until the absorbance reached 0.7 to 1.0 and the dilution ratio was recorded.

#### Lignin: Acid-insoluble lignin

The glass crucible and the acid insoluble residue were burned in a muffle furnace at 575°C for at least 3 h at a heating rate of 10°C/min until all organic matter turned into ash. After ashing, the crucible was cooled to room temperature in a dryer following which the crucible and ash content were weighed.

The mass percentage of acid-soluble lignin was calculated as follows:

$$ASL=\frac{A\times V\times N\times w1\times L}{wef\times w0\times \varepsilon }$$

where, *ASL*: acid-soluble lignin in the sample, mass percent %; *A*: the average of absorbance value at 320 nm in the filtrate; *V*: the volume of the filtrate, 87 mL; *N*: the dilution ratio of the filtrate; *L*: the thickness of the cuvette (cm); *wef*: the quantity (g) of the sample for two-step acid hydrolysis; *w0*: the quantity (g) of the sample before extraction; w1: the quantity (g) of the sample after extraction; *ε*: the absorptivity % of acid-soluble lignin at 320 nm, 30 L/(g.cm) for cornstalk and 25 L/(g.cm).

The mass percentage of acid-insoluble lignin was calculated as follows:

$$AIL=\frac{[\left(m2-m1\right)-(m3-m1)]\times w1}{wef\times w0}\times 100$$

where, *AIL*: acid-insoluble lignin in the sample, mass percent %; *m1*: the quantity (g) of the crucible with glass sand core; *m2*: the quantity (g) of the crucible with glass sand core and acid-insoluble residue; *m3*: the quantity (g) of the crucible with glass sand core and ash. This measurement was performed in triplicate (n = 3).

#### Hemicellulose

The filtrated hydrolysate (1 mL) was mixed with 2 mL of solution A and 0.134 mL of solution B, boiled for 20 min, and cooled to room temperature. Solution A was prepared by dissolving 0.1 g FeCl_3_ in 100 mL 37% HCl, while solution B was prepared by dissolving 6 g orcinol in 100 mL of absolute ethanol. Absorbance was measured at 660 nm, and the sugar content was calculated using the xylose standard curve, multiplied by the coefficient, 0.9. This process was performed in triplicate (n = 3).

#### Cellulose

The filtrated hydrolysate (1 mL) was mixed with 2 mL anthrone solution, boiled for 5 min and cooled to room temperature. Anthrone solution was prepared by dissolving 0.2 g anthrone in 100 mL concentrated sulfuric acid. Absorbance was measured at 620 nm, and the sugar content was calculated using the glucose standard curve, multiplied by the coefficient, 0.9. The procedure was performed in triplicate (n = 3).

### 2.10. Model sample

The human cell line, *OE33* (ZEYE^®^), was cultivated as a model sample for DNA extraction. The complete culture medium included 89% RPMI-1640 basal medium (GENVIEW^®^), 10% fetal bovine serum (GIBICO^®^) and 1% Penicillin-Streptomycin Solution (HYCLONE^™^). The cell culture was washed thrice using PBS (SOLARBIO^®^) before use.

### 2.11. Adjustment of crystallinity

Bamboo paper (BP) and degreased cotton (DC) were heated in an electro-thermostatic blast oven (JINGHONG DHG-9240) at 180°C for 0, 15, 30, 60 and 120 min to serially alter the crystallinity of both adsorbents without changing their components [47]. Treatment started when the temperature reached 180°C. Treated adsorbents were applied in DNA extraction. Each process was performed in triplicate (n = 3).

### 2.12. The quantity, quality and integrity of extracted DNA

A 1 μL sample was added to the sampler of a Nanogenius photometer (MAPADA) to measure concentration and purity (OD260/OD280 and OD260/OD230 ratios) of the extracted DNA under UV illumination. Elution buffers used in each condition acted as the control. Each procedure was performed in triplicate (n = 3).

Agarose gel was prepared using 1% (w/v) agarose (VAKE) in 1% TAE buffer (SOLARBIO^®^). Gel electrophoresis was run for 30 min at 100 V in an electrophoresis apparatus set (DYY-7C LIUYI). The gel image was then acquired using an ImageQuant LAS 500 under UV illumination. The electrophoresis marker was λEcoRI Hindlll (MEI 5BIO). Five microliters of marker and samples mixed with 1 μL SYBR Green I (working solution contains: 1 μL SYBR Green I <10000 x> <SOLARBIO^®^>, 1 mL 6 x Loading buffer <SOLARBIO^®^> and 1 mL 1% TAE buffer <SOLARBIO^®^>) respectively, were loaded into agarose gel for electrophoresis. Each procedure was performed in triplicate (n = 3).

### 2.13. Polymerase Chain Reaction (PCR)

PCR was performed on DNA extracted from the model sample using the innovative protocol and current approaches in the assembled extraction spin column set. Human GAPDH was selected as the target sequence. Amplification was performed in a BIONEER Exicycler^™^96. Synthesized primers (BGI), amplification conditions and dosage proportions are shown (Table 3). The amplified PCR products were identified via electrophoresis using a 2% (w/v) agarose (VAKE) gel in a 1% TAE buffer (SOLARBIO^®^). M5 DL2000 (MEI 5BIO) was used as the electrophoresis marker. For other parameters, refer to Part 2.12. Each procedure was performed in triplicate (n = 3).

**Table 3:** PCR reaction details.

**1.PRIMER**		
	**Sequences (5´-3´)**	**Product size (bp)**
**forward**	GAACGGGAAGCTCACTGG	123 bp
**reverse**	GCCTGCTTCACCACCTTCT	
**2.REAGENTS**		
	**Final quantity**	**Volume**
**ddH2O**	accordingly,	~5.8 μL
**DNA template**	0.1 μg	~1.0 μL
**primer**	0.8 μM	3.2 μL
**Easy-Load**^**™**^ **PCR Master Mix**	1X	10.0 μL
**total volume**	/	20.0 μL
**3. STEPS**		
	**Temperature**	**Time**
**pre-denaturation**	94°C	3 min
**denaturation**	94°C	30 s
**annealing**	55°C	30 s
**elongation**	72°C	10 min
**cycles**	30	/

## 3. Results and discussion

### 3.1. Current status of DNA extraction approaches

In the previous sections, we described the status of current DNA extraction approaches. Five common brands of commercial kits covering currently used methods were selected. The quantity of extracted DNA, changes in quantity after replicating elutions, and purity ratios of OD260/OD280 and OD260/OD230 were analyzed.

**Fig 1:**
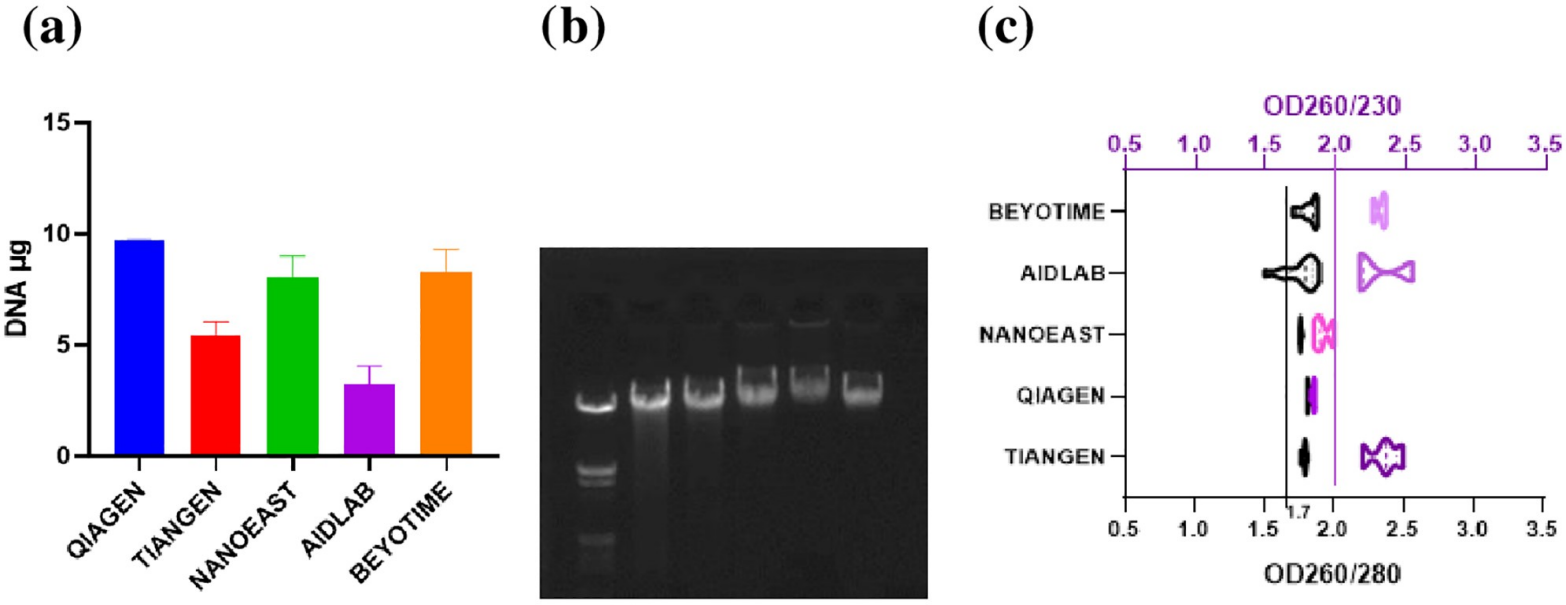
(a) Comparison of DNA extracting capacities of five current methods. (b) DNA electrophoresis of the related samples (Marker, QIAGEN, TIANGEN, NANOEAST, AIDLAB, BEYOTIME). (c) The OD260/OD280 (black) and OD260/OD230 (purple) purity ratios of the related samples.

**Fig 2:**
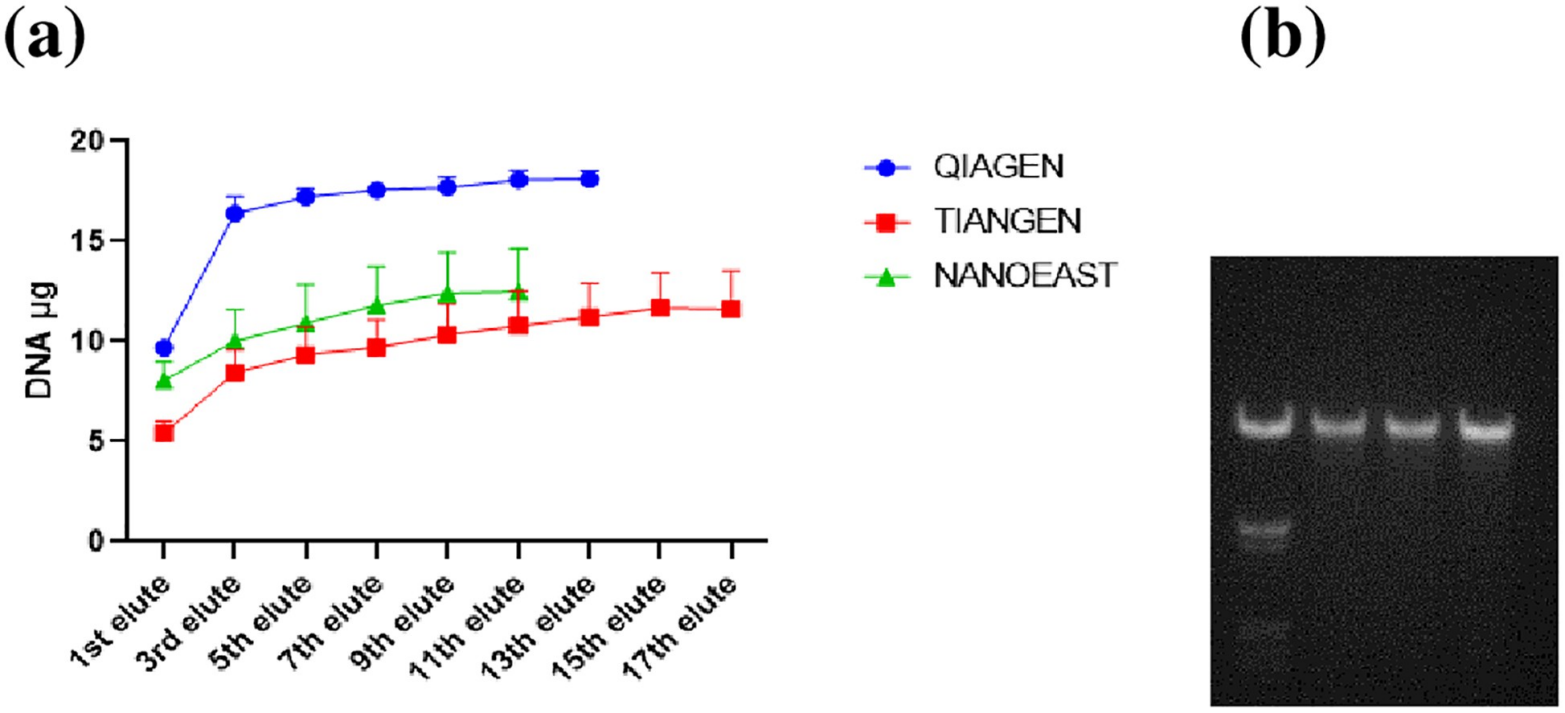
(a) The amount of extracted DNA during repeated serial elutions among TIANGEN, NANOEAST and QIAGEN. (b) DNA electrophoresis of the related samples (Marker,TIANGEN, NANOEAST and QIAGEN).

Among the current approaches tested, QIAGEN showed the highest DNA extracting capacity with consistent electrophoresis results (Fig 1a & 1b). Therefore, a newly developed method which achieves an equal or higher DNA extraction, comparative purity and other benefits (i.e., processing, economic etc.), would be considered as more successful. The amount of extracted DNA increased with increasing elution time, where relatively large increases were observed between the first and third elution while subsequent increase was gradual until equilibrium was reached (Fig 2a & 2b). For routine DNA extraction using CC and MB methods, triple elution should be considered, especially for experiments that focus on the amount of DNA extracted. Besides the amounts extracted, the OD260/OD280 ratio of QIAGEN, TIANGEN and NANOEAST fell between 1.7 and 2.0 (Fig 1c) while a ratio close to, or less than, 1.7 was detected for BEYOTIME and AIDLAB. The OD260/OD230 ratios of NANOEAST and QIAGEN were less than 2.0 while those of BEYOTIME, AIDLAB and TIANGEN fell between 2.0 and 2.6 (Fig 1c). When both purity indexes were comprehensively considered, only TIANGEN and BEYOTIME passed. Falling within the CC and MB methods, QIAGEN, TIANGEN and NANOEAST were selected for comparison in the experiments that were to follow. The toxic solution based BEYOTIME (chloroform) and AIDLAB approaches were excluded. The statuses of the current DNA extraction approaches are summarized (Table 4 & S4 Fig).

**Table 4:** Summary of the current approaches.

Brand	QIANGEN	TIANGEN	NANOEAST	AIDLAB	BEYOTIME
**Phase of medium**	Solid	Solid	Solid	Liquid	Liquid
**Adsorbent**	Silica-based membrane	Silica-based membrane	MBs	NA	NA
**Proteinase K**	Yes	Yes	Yes	Yes	Yes
**Harmful reagents**	No	No	No	Yes	Yes
**Heating**	optional	Yes	Yes	Yes	Yes
**Operating time** ^1^	~30 min	~30 min	~45 min	>1 h	Overnight
**Cost ($)**	276/50T	67/50T	73/50T	67/50T	28/50T
**OD260/OD280**	1.8–1.9	1.7–1.9	1.7–1.8	1.5–2.0	1.7–2.0
**OD260/OD230**	1.8–1.9	2.2–2.5	1.8–2.0	2.2–2.6	2.2–2.4
**Size of DNA**	~ 21 kb	~ 21 kb	~ 21 kb	~ 21 kb	~ 21 kb

^1^Only the operation time mentioned in the protocols were counted (e.g., centrifugation and water bath), and buffer filling time was excluded.

### 3.2. The representative PF-BAs, BP and DC, showed high suitability of DNA extraction

Whatman^™^ Grade 1, Grade 3 and GF/F filters were the commercial filter papers selected to be tested for their DNA extracting capacity. An analysis of the similarities between these filter papers (Whatman^™^ Grades 1 and 3, except GF/F) revealed that their appearance and texture were similar to those of papers used daily. On one hand, paper is usually manufactured using wood as the raw material, and thus it is likely that these filters were also made using wood fiber as the main material. On the other hand, cellulose constitutes the polymer base of filter paper [32, 33]. Wood fiber is a type of plant fiber. Therefore, if filter papers made from wood fiber demonstrate noteworthy DNA extraction capacity, then PF-BAs made from plant fiber should also exhibit considerable DNA extraction capacity. Apart from the above mentioned commercial filter papers, no other PF-BAs were studied for the current DNA extraction study. Therefore, this stage of the experiment explored the capacity of the new PF-BAs, WP, SP and BP, to extract DNA with the existing protocol (TIANGEN). The results indicated that BP showed superior potential as an adsorbent that can be used for DNA extraction purposes, in terms of both quantity and quality.

**Fig 3:**
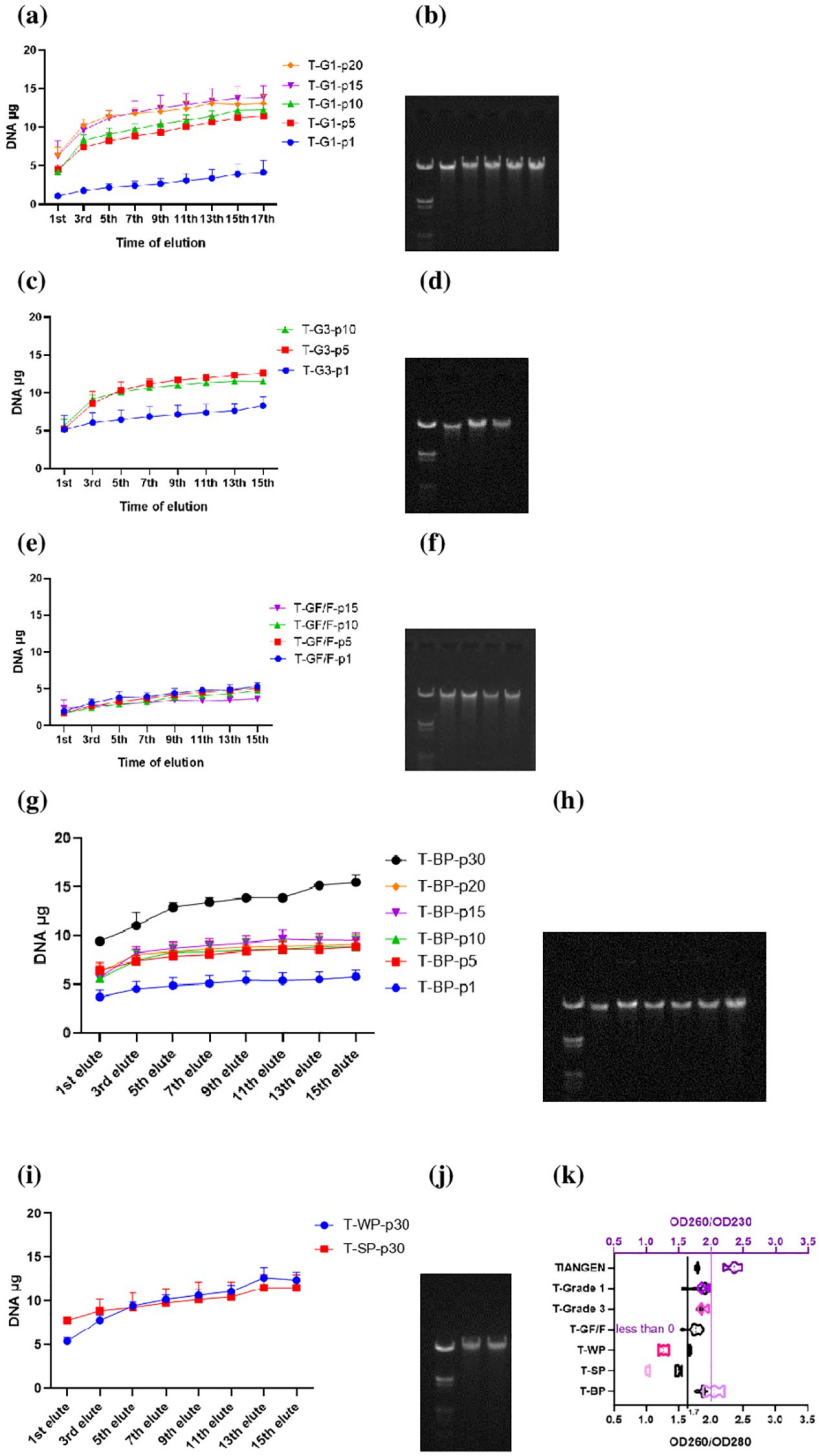
Comparison of the DNA extracting capacities of selected plant fiber-based DNA adsorbents, using the TIANGEN protocol. (a): The quantiy of extracted DNA in different pieces of Whatman^™^ Grade 1 filter paper. (b): DNA electrophoresis of related samples (Marker, *T-Grade1-p1, -p5, -p10, -p15, -p20). (c): The quantity of extracted DNA in different pieces of Whatman^™^ Grade 3 filter paper. (d): DNA electrophoresis of related samples (Marker, T-Grade3-p1, -p5, -p10). (e): The quantity of extracted DNA in different pieces of Whatman^™^ GF/F filter paper. (f): DNA electrophoresis of related samples (Marker, T-GF/F-p1, -p5, -p10, -p15). (g): The quantity of extracted DNA in different pieces of BP. (h): DNA electrophoresis of related samples (Marker, T-BP-p1, -p5, -p10, -p15, -p20, -p30). (i): The quantity of extracted DNA in 30 pieces of WP and SP. (j): DNA electrophoresis of related samples (Marker, WP, SP). (k): The OD260/OD280 (black) and OD260/OD230 (purple) purity ratios of related samples. *T and p refers to TIANGEN protocol and the number of the adsorbent pieces used, respectively.

The quantity of extracted DNA increased with increasing elution time, where relatively large increases were captured between the first and third elution, while the subsequent increase was gentle until equilibrium (Fig 3a–3j). The quantity of extracted DNA increased with increasing numbers of adsorbent pieces, where Whatman^™^ Grades 1 and 3 showed their upper limit at 15 (Fig 3a & 3b) and 5 pieces (Fig 3c & 3d), while less DNA extraction capacity was observed in Whatman^™^ GF/F (Fig 3e & 3f). BP showed the highest DNA extracting capacity among the selected adsorbents (Fig 3g & 3h). WP and SP also showed relatively strong capacities (Fig 3i & 3j), comparable to Whatman^™^ Grade 1 and 3. The purity ratio OD260/OD280 of BP, Whatman^™^ Grade 3 and most of Whatman^™^ Grade 1 fell between 1.7 and 2.0 (Fig 3k). Among these four, the OD260/OD230 ratio of Whatman^™^ Grade 1 and 3 fell between 1.8 and 2.0, while those of TIANGEN and partial BP were above 2.0 (Fig 3k).

The results (Fig 3a–3k) of the current study demonstrated that the DNA extraction capacity of BP was approximately twice that of the current approach (using TIAGEN protocol), indicating that BP was highly suitable for DNA extraction. BP is mainly a combination of bamboo fiber, which, in turn, is made of natural cellulose ((C_6_H_10_O_5_) _*n*_), a polymer with six hydroxyl groups (-OH) per repeat unit. Presumably, plant fiber plays a key role in the ability of PF-BAs to adsorb DNA. As the natural cellulose content in cotton is known to be about 90% [48], DC was included in this experiment as a new potential PF-BA suitable for DNA extraction. We then applied the QIAGEN protocol to BP, DC and adsorbents from current approaches to further confirm the suitability of BP and DC, the new PF-BAs, for DNA extraction, and compared the performance of different types of adsorbents.

**Fig 4:**
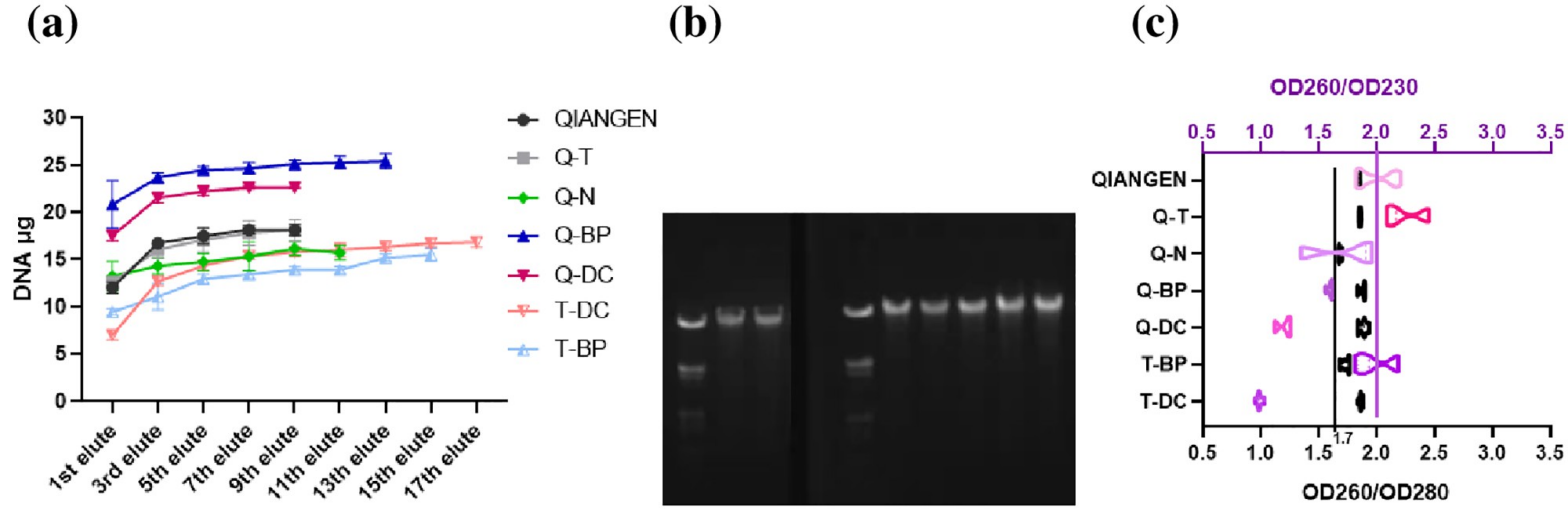
(a) Comparison of DNA extracting capacity of BP, DC and three types of adsorbents from kits using QIAGEN and TIANGEN protocols. (b) DNA electrophoresis of related samples (Marker, *T-DC, T-BP, marker, QIAGEN, Q-NANOEAST, Q-TIANGEN, Q-DC & Q-BP). (c) The OD260/OD280 (black) and OD260/OD230 (purple) purity ratios of related samples. *T-, Q-, -T and -N refer to TIANGEN protocol, QIAGEN protocol, TIANGEN adsorbent and NANOEAST beads, respectively.

The performance of BP and DC under the QIAGEN protocol was approximately two times better than that under the TIANGEN protocol (Fig 4a & 4b). This indicated that the QIAGEN protocol provided a more suitable extracting system for these two adsorbents. BP and DC showed significantly higher DNA extracting capacities (p<0.01) than those of silica-based and MB adsorbents via the QIAGEN protocol (Fig 4a & 4b), demonstrating that BP and DC were highly suitable to act as new PF-BAs for DNA extraction. The highly overlapping data lines of QIAGEN and Q-T (Fig 4a & 4b) indicated that those adsorbents claimed to be silica-based by the two companies may not be essentially different. The purity ratio OD260/OD280 of BP, DC, TIANGEN and QIAGEN, except MB (applying QIAGEN protocol), ranged between 1.7 and 2.0 (Fig 4c). The purity ratios of OD260/OD230 of QIAGEN and TIANGEN ranged between 1.8 and 2.5 while in the cases of the adsorbents, MB, BP and DC, the ratio ranged from 1.1 to 1.9 (Fig 4c). This demonstrated that in order to achieve the suggested purity levels, a specific extracting system involving lysis, washing and elution buffers need to be established for BP and DC in DNA extraction [30, 40, 41].

The fact that QIAGEN protocol performed better than the TIANGEN one for BP and DC, indicated that a protocol even more suitable for these two new PF-BAs may exist. BP and DC showed significantly higher quantities of extracted DNA than those of the current approaches that used the same protocols (TIANGEN and QIAGEN). This indicated that two directions were worthy of further exploration. Firstly, the properties of PF-BAs associated with their remarkeable DNA adsorption capacities must be determined. Secondly, although the related OD260/OD280 ratio falls within the satisfactory range of 1.7 to 2.0, the related OD260/OD230 ratio cannot be considered as satisfactory, indicating the existence of impurities, such as polysaccharides, salts and/or phenolic compounds. Thus, the development of a suitable extracting system for BP and DC is deemed necessary.

### 3.3. The characteristics of PF-BAs

**Fig 5:**
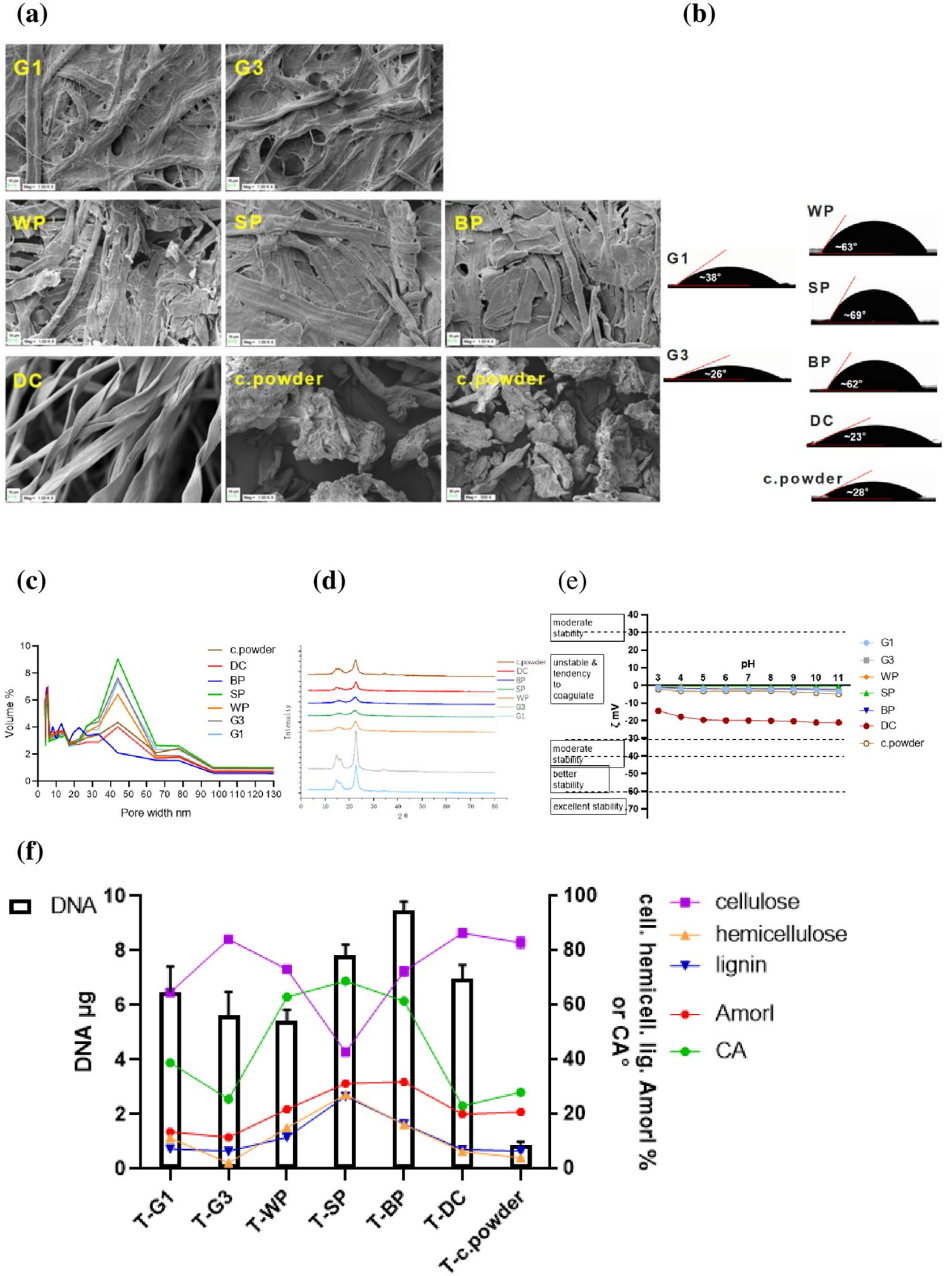
(a) Images of related PF-BAs under a scanning electron microscope (SEM) (x1000). (b) Contact angle of related PF-BAs. (c) Distribution of pore size in the selected PF-BAs using BJH model. (d) Crystallinity of related PF-BAs assessed via XRD analysis. (e) Zeta point (pH 3–11) of related PF-BAs (c.powder refers to cellulose powder). (f) Comprehensive histogram and line chart visualizing the relationship among the quantities of extracted DNA, cellulose, hemicellulose, lignin, AmorI and CA in the selected adsorbents.

The costs of BP and DC, if used as the new DNA PF-BAs would be 0.065 USD/m^2^ and 0.0057 USD/g which are minimal because they are 415 and 4737 times lower than the average price of the selected Whatman^™^ G1 and G3 filter papers (25 and 29 USD/m^2^, respectively) (Table 1). A Zeta potential within ±30 mV is usually identified as the dividing line for judging the stability of the target, because targets that fall within this range are regarded as showing coagulation or a tendency to coagulate (surface or particle). The Zeta potential of the selected PF-BAs (including BP and DC) fell within ±20 mV (Fig 5e) indicating that the surface potential of PF-BAs was weak [49, 50]. SEM graphs allow the adsorbents to be microscopically and intuitively viewed (Fig 5a). Visually, these adsorbents are divisible into four categories as follows: (i) the fiber bundles of G1 and G3 that are attached to the “adhesive” surface, via deep reaction and long stirring activities, showing obvious integrity; (ii) the distinct fiber bundles of WP, SP and BP are integrated, interlaced and stacked as fiber bundles without obvious rules of arrangement; speculatively, they were processed at a lower degree than (i); (iii) simply degreasing and washing makes the fibers of DC become loose and clear, similar to that seen in the original state of cotton; (iv) acid treatment, degradation, washing, filtering, drying and grinding allowed the micromorphology of c.powder (cellulose powder) to be viewed as particles (90 μm) under SEM.

C. powder indicates that the microstructure is a key feature of DNA PF-BAs. C. powder shares a similar composition (cellulose, hemicellulose and lignin), AmorI and CA with DC (Fig 5f). Although CA showed no obvious changes in hydrophilicity, powdering greatly reduced the water retainability of c.powder. The very low DNA extracting capacity of c.powder could only be due to the change from a fibrous structure to 90 μm sized particles, leading to a loss of capillary force required for its siphonic effect, which greatly reduced the capacity to capture DNA. Considered together, this conversely proved that the fibrous structure of PF-BAs was the core factor that determined DNA adsorption on PF-BAs. Based on the above (Part 1.4), these adsorbents may more appropriately be considered as PF-BAs rather than cellulose-based ones [32, 33, 35] since the fibrous structure of plants is the established prerequisite for such adsorption. CA of the hydrophilic adsorbent falls between 0° to 90° indicating that the smaller the CA the stronger the hydrophilicity (Fig 5b). All selected adsorbents were hydrophilic, among which DC showed the strongest hydrophilicity. The pore size distribution of the PF-BA ranged from 30 to 65 nm in diameter, which enables them to be considered as mesopores and macropores. The diameter of DNA is 2 nm [51] indicating that the interaction between DNA and the PF-BAs was based on surface interaction and not on the pore size of adsorbents, as surface interaction is probably the only reason that retains DNA on PF-BA, because otherwise DNA would easily pass through the meso/macropores (Fig 5c) while being centrifuged for the purpose of extraction.

The comprehensive histogram and the line chart in combination with correlation related values, indicated that only AmorI was highly correlated (r = 0.078; p = 0.065) with the quantity of DNA extracted by the PF-BAs where the p-value (0.065) was close to being significant at the 0.05 level (Fig 5b–5e; Table 5). The other factors showed weak or no correlation with the quantity of extracted DNA, which may be attributable to differences between adsorbents caused by differences between the various characteristics of each adsorbent. For example, the compositions of cellulose, hemicellulose and lignin in G1 and BP are totally different, further to which they are three times different from that of AmorI, thereby affecting the outcome of CA. Subsequent results (Part 3.5) showed that the quadruple times more efficient nucleic acid extraction from bamboo paper and cotton was highly correlated with their crystallinity and hygroscopicity.

**Table 5:** Degree of linear fitting and correlation among the extracted DNA, cellulose, hemicellulose, lignin, AmorI, and CA in the adsorbents.

	Coefficient of determination	Correlation coefficient		Correlation
	R^2^	|r|	P value	
**CA vs. DNA**	0.14	0.41	0.43	Weak or no
**AmorI vs. DNA**	0.52	0.78	0.065	Relatively high
**Cellulose vs. DNA**	0.084	0.32	0.54	Weak or no
**Hemicellulose vs. DNA**	0.20	0.49	0.33	Weak or no
**Lignin vs. DNA** ^1^	0.27	0.57	0.24	Weak or no

^1^c.powder was excluded from the linear fitting and correlation calculations due to its relevant values being completely different from those of the other samples (Fig 5f).

As the relatively more affordable BP and DC showed significantly higher DNA extracting capacities, their specific DNA extraction system was developed to optimize the quality and quantity of extracted DNA. Furthermore, an analysis of the degree of linear fitting and correlation was performed on each adsorbent to explore the principles underlying such adsorption.

### 3.4. The quadruple efficient DNA extracting system with PF-BAs (BP and DC)

Details pertaining to the contents and composition of DNA extraction commercial kits are trade secrets that remain the intellectual property of the kit makers, making any analysis of the protocols pertaining to the composition of commercial buffers very difficult as well as unrealistic.

Due to the above situation, this study referred to the relevant publicly available and authoritative formulations as basic recipes, and optimized them for use with new DNA PF-BAs. Hence, one each of ionic detergent based [30] and non-ionic detergent based [32] lysis buffers, respectively, were selected. The pH of the processing buffers were similar to that of QIAGEN and TIANGEN, because they were both produced from an overall relatively stronger acidic lysis condition, which was subjected to slight acidic washing steps to reach alkiline elution. By contrast, the buffer solutions of the MB method were basically maintained at the neutral level (Table 6). Both lysis buffers, with their pH serially adjusted for DNA extraction, were applied to obtain a pH suitable for sample lysis and DNA binding to these PF-BAs. Although the composition of the washing buffer is presumably confidential, it was apparent that existing commercial kits invariably use different percentages of ethanol solution as the washing buffer for DNA extraction (Table 6). These washing buffers contain 56.7% to 70% (v/v) ethanol. Because ethanol also acts as a DNA separating solution that separates DNA from water, the adsorption of DNA on PF-BAs is achieved by relatively dissolving DNA in water and undissolving it in ethanol. Based on this property, it is assumed that a higher concentration of ethanol solution within a certain range may theoretically induce more DNA to remain attached to the adsorbent during the washing process, thereby ensuring that as much DNA as possible is extracted at the final elution. Thus, 70% ethanol was selected as the washing buffer. The recipe for the elution buffer was the only available one on the QIAGEN website [43]. The specific scheme is shown in the methods section.

**Table 6:** pH of the serial buffer solutions and ethanol used in the existing DNA extraction protocols of current approaches.

EB^1^	WB2	WB1	LB2	LB1		DNA separating solution	Starting volume	Absolute ethanol added	Ethanol (v/v)
					pH				
8.22	6.41	4.65	5.06	8.4	**TIANGEN**	Absolute ethanol	GD-13 mL	17 mL	56.7%
							PW-15 mL	60 mL	80.0%
8.93	6.87	6.10	\	5.59	**QIAGEN**	Absolute ethanol	AW_1_-19 mL	25 mL	56.8%
							AW_2_-13 mL	30 mL	69.8%
7.54	7.00	7.00	7.88	7.97	**NANOEAST**	Absolute isopropanol	/	/	70.0%

^1^LB: Lysis buffer, WB: Wash Buffer, EB: Elution Buffer

**Fig 6:**
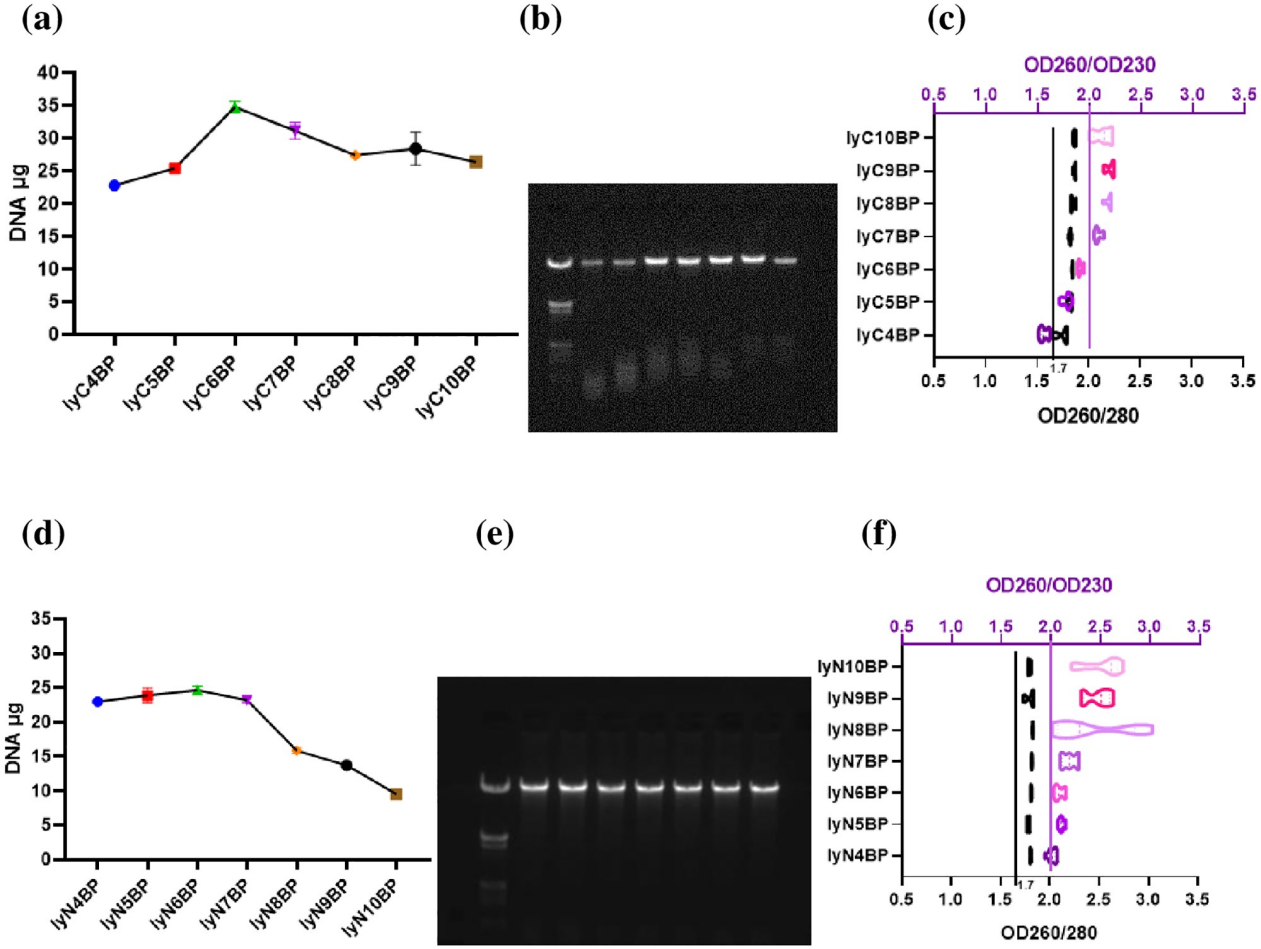
(a) The capacities of lysis buffer C (pH 4 to 10), and elution buffer Q (pH 9) for extracting DNA on BP, using the new protocol. (b) DNA electrophoresis of the related samples (Marker, *lyC4,5,6,7,8,9,10BP; *lyC and the number refers to lysis buffer C and the pH of lysis buffer C). (c) The OD260/OD280 (black) and OD260/OD230 (purple) purity ratios of the related samples. (d) The capacities of lysis buffer N (pH 4 to 10), and elution buffer Q (pH 9) for extracting DNA on BP, using the new protocol. (e) DNA electrophoresis of the related samples (Marker, *lyN4,5,6,7,8,9,10BP; *lyN and the number refers to lysis buffer N and the pH of lysis buffer N). (f) The OD260/OD280 (black) and OD260/OD230 (purple) puriy ratios of the related samples.

Lysis buffer N (pH 4–7) showed high suitability for DNA extraction on BP and DC (Fig 6d & S6a Fig). DNA electrophoresis on BP and DC using lysis buffer C, generated small DNA fragments of approximately 500 bp as indicated by the commercial marker. Although lysis buffer C showed a relatively higher capacity for DNA extraction, DNA electrophoresis generated undesired DNA fragments (Fig 6a & 6b; S5a & S5b Fig), indicating the unsuitability of lysis buffer C and its current composition for DNA extraction. On the contrary, lysis buffer N (pH 4–7) with BP and DC as DNA PF-BAs yielded remarkably improved DNA extraction results that were approximately 2.5 times larger than that yielded by the QIAGEN kit. All or most related OD260/OD280 and OD260/OD230 ratios were associated with satisfactory purities (Fig 6e & S6c Fig), indicating that the extracted DNA was sufficiently pure to be used in most downstream experiments [30, 40, 41]. The new method of DNA extraction on PF-BAs using lysis buffer N requires further optimization of the elution buffer.

As lysis buffer N (pH 5 to 7) showed high suitability for DNA extraction on BP and DC (Fig 6d–6f & S6 Fig), the suitability of the pH of elution buffer Q, ranging from 6 to 10, was explored for the optimized pair of lysis buffer N and elution buffer Q.

**Fig 7:**
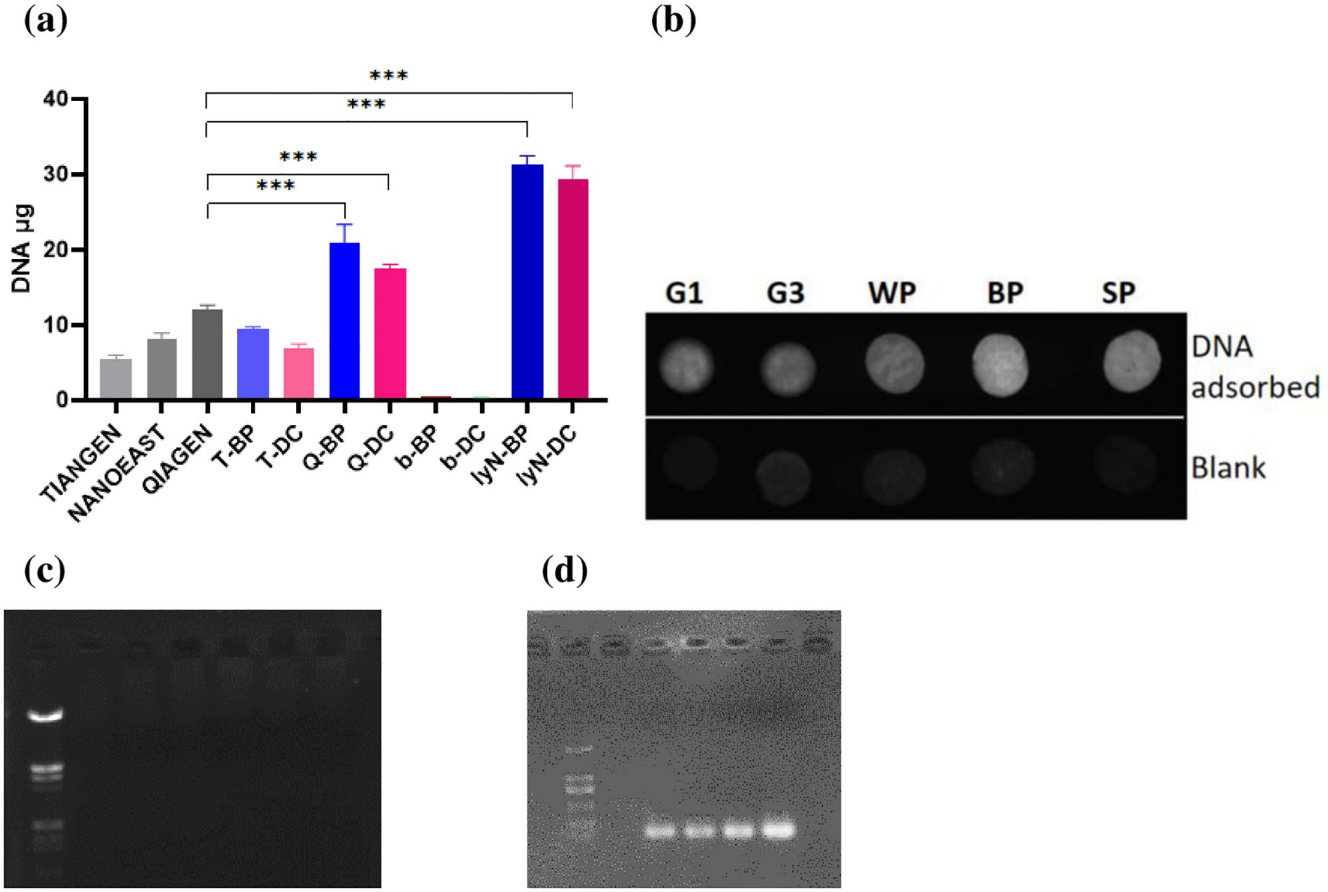
(a) Comparison of the DNA extracting capacities of the current approaches, BP/DC with commercial protocols, and BP/DC with the new protocol. (b) A UV imager was used to compare DNA absorbed utilizing a single piece of PF-BA against blank controls. (c) DNA electrophoresis of the blanks of BP/DC with the new protocol (Marker; BP<n = 3>; DC<n = 3>). (d) The PCR products of negative control, TIANGEN, NANOEAST, QIAGEN and DC.

The lysis buffer N (pH 5–7) paired with elution buffer Q (pH 8–9), extracted an ideal quantity of stable DNA, while the elution buffer Q with a pH over 9 (pH = 10) and less than 7 (pH = 6) reduced DNA extraction (S7a, S7c and S7d & S9 Figs). Mild acidic conditions (pH 5–7) favored the binding of DNA to BP and DC while the mild basic eluent dissociated DNA from the two (S9 Fig). The average quantity of DNA extracted on BP and DC was 5.5–5.8 times over that achieved via TIANGEN, 3.7–3.9 times over that achieved via NANOEAST, and 2.5–2.6 times over that achieved via QIAGEN DNA extraction (Fig 7a). Thus, the new plant fiber-based DNA adsorbents, BP and DC, are quadruple times more powerful than the current commercial ones. Improving efficiency quadruple times in such a manner indicates that targeted DNA extraction may lead to quadruple-times improved accuracy and reliability of related downstream analysis and detection. Furthermore, BP showed slightly higher extraction capacity than DC. The related blanks did not exert a visible effect on extraction (Fig 7a & 7c). The results of the UV image of a single disc of PF-BA with DNA adsorbed, compared to the blanks, were consistent with those of related digital data (Figs 7a & 7b; 3a, 3c and 3i). Moreover, the electrophoresis of related PCR products demonstrated that GAPDH was amplified indicating the feasibility of molecularly diagnosing extracted DNA downstream (Fig 7d). Thus, DC and BP appear to be able to act as the new PF-BAs, which are capable of extracting DNA quantities that approximate quadruple-times that of currently existing approaches.

After optimizing DNA extracting conditions for PF-BAs, represented by BP and DC, as shown above, it becomes important to confirm the key characteristics of PF-BAs that are correlated with the efficiency of DNA extraction. Together these two factors may guide future studies from the perspective of different disciplines.

### 3.5. Crystallinity and hygroscopicity of PF-BAs were tightly correlated with the efficiency of DNA adsorption

Crystallinity of the two PF-BA representatives, BP and DC, was serially modified under 180°C for 0, 15, 30, 60 and 120 min without changing their composition [26]. The comprehensive histogram and line chart exhibiting the degree of linear fitting and correlation demonstrated that AmorI and CA were highly correlated (|r| = 0.90 to 1.00) with the efficiency of BP and DC for extracting DNA. Further, the correlation between BP and AmorI was very close to being significant (p = 0.057) at the 0.05 significance level (S8c, S8d, & S8e Fig; Table 7). AmorI was positively and CA was negatively highly correlated with the efficiency of both BP and DC for DNA extraction. The AmorI of serially heated BP and DC fell consecutively from 31.8% to 27.5%, and 20.0% to 16.0%, (S8a & S8c Fig) while their CA value went up from 61.46° to 78.43°, and 23.06° to 65.28° (S8a & S8d Fig), respectively. AmorI and CA are two important indexes that measure the availability of hydrogen bonds in BP and DC as well as their hygroscopicity. Increased CA was accompanied by a decrease in the AmorI of both BP and DC. The very high correlation between the two indexes and the extraction efficiency of DNA essentially reflected the very high correlation between the degree of amorphousness as well as hygroscopicity in PF-BAs, and the extraction efficiency of DNA. Because of this high correlation, these two indexes act as significantly important guidance for the study of PF-BAs in the future.

**Table 7:** Degree of linear fitting and correlation among the extracted DNA, AmorI, and CA in BP and DC.

	Coefficient of determination	Correlation coefficient		Correlation
	R^2^	|r|	P value	
**BP vs. CA**	0.87	0.93	0.022*	Very high
**BP vs. AmorI**	0.75	0.87	0.057	High
**DC vs. CA**	0.89	0.95	0.015*	Very high
**DC vs. AmorI**	0.82	0.91	0.034*	Very high

Although AmorI is proportional to DNA adsorption on PF-BA, the higher the AmorI the higher the swelling of PF-BA, thereby reducing the toughness of PF-BA. If the toughness of PF-BA drops to a certain extent, PF-BA will be damaged, which will result in detachment during extraction, thereby affecting the purity of extracted DNA. The same applies to hygroscopicity analysis. Therefore, optimal PF-BA may be predicted using the status of balance between the two indexes, amorphous region and hygroscopicity, rather than one being simply larger or smaller.

Hydrogen bonding [52] has been proposed as the main factor that mediates the adsorption of DNA on PF-BAs, such as BP and DC. BP and DC are made of bamboo and cotton fibers which are microstructurally composed of crystalline and amorphous regions that are interlaced without clear boundaries [53] and chemically bear six “-OH” per cellulose repeating units. The crystalline region is arranged in a tight and well-ordered manner via inner intermolecular hydrogen bonds, making it resistant to water and reactions, thereby maintaining the strength of PF-BAs. By contrast, the amorphous region displays a disordered fibrous-like structure which forms a relaxed irregular network that is easily accessible to water and bears hydrophilic groups (mainly “-OH”), facilitating adsorption [53].

These results demonstrated that AmorI is proportional to the adsorption efficiency of DNA on PF-BAs, such as BP and DC (S9 Fig). Also, the dominance of meso/macropores in PF-BAs indicated that surface interactions were the main factors facilitating DNA adsorption on PF-BAs (Part 3.3). Cellulose is the unique and single component that is responsible for the crystalline structure arrangement seen in PF-BAs, where this arrangement is due to intermolecular hydrogen bonding between hydroxyl groups [53]. Proper heating physically transforms amorphous cellulose into crystalline cellulose in PF-BAs (S8a & S8c Fig), essentially reducing available hydroxyl groups in the amorphous region to unavailable hydroxyl groups in the crystalline region by occupying the available hydroxyl groups via the formation of intermolecular hydrogen bonds. This unique reduction resulted in a drop in the quantity of extracted DNA (S8c Fig), indicating that the interaction between hydroxyl groups and DNA had decreased. Furthermore, the results of this study showed that the availability of hydroxyl groups, represented by AmorI, was found to be strongly and positively correlated with the efficiency of DNA extraction (Table 7) by the serial treated PF-BAs, represented by BP and DC. Moreover, “-OH” is stable and does not ionize at all in the cellulose polymer [54]. At appropriate pH, hydrogen bonds are formed between available “-OH” from the amorphous region of PF-BAs and DNA, leading to this specific adsorption.

By contrast, adsorption on CC and MB depends on the cation bridge built between the negative silanol groups and DNA (S1 Fig) on account of “-OH” being unstable in silica-based material [54]. Cations are also considered as contributors to the adsorption between PF-BA (Whatman^™^ Grade 1) and DNA, because these cations neutralize the negative charges on both [33]. However, the contribution of cations may not be entirely necessary for adsorption between PF-BA and DNA, as evidenced by the new PF-BA based extraction method which is quadruple-times more effective than the current approaches, although its lysis buffer N does not contain any cations (usually Na^+^).

In summary, (i) plant fibrous structure is a prerequisite for effective adsorption of DNA on PF-BA; (ii) lysis buffer (pH 5–7) paired with elution buffer (pH 8–9), may lead to ideal DNA extraction by PF-BAs, such as BP and DC, which extract quadruple-times the DNA extracted by the current approaches; (iii) hydrogen bonding is the main contributor to this specific adsorption; (iv) the two indexes, amorphous degree (AmorI) and hygroscopicity, of PF-BAs, warrant serious enquiry in any studies investigating PF-BAs in the future. The findings of this study may be applicable for other extractions involving nucleic acids with similar biochemical characteristics [3].

### 3.6. The correlation model of DNA extraction with PF-BAs

**Fig 8:**
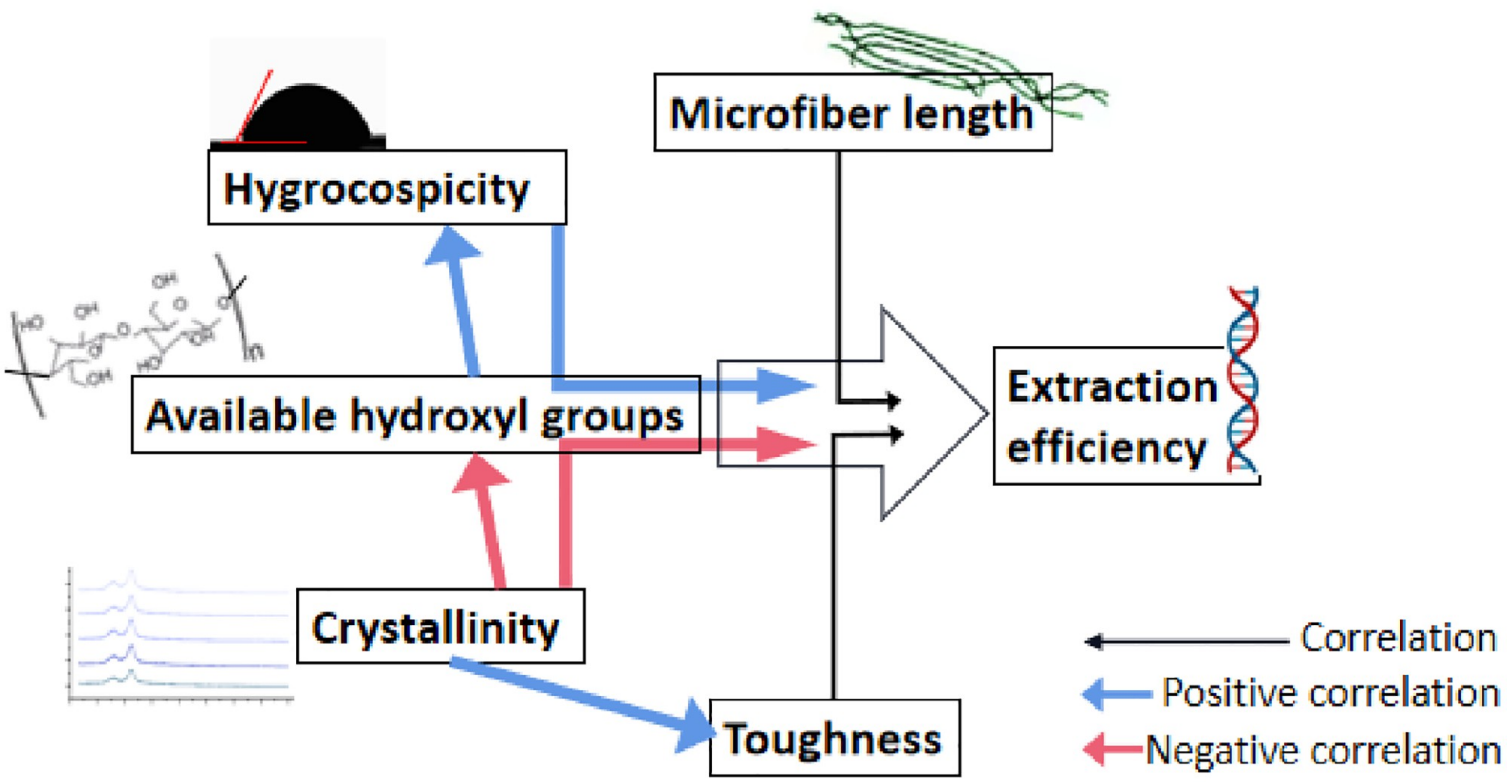
The correlation model.

The correlation model demonstrated that the adsorption material based on plant fibers may be broadly used for purposes of nucleic acid adsorption and extraction. The results of this study showed that plant fiber adsorbents show higher efficiency in nucleic acid adsorption and extraction than current approaches. This efficiency is closely related to the number of “free” hydroxyl groups, which, in tun, is determined by the degree of crystallinity of the plant fiber adsorbent, and its water absorption capability, based on the act of siphoning. Firstly, the hygroscopicity of a plant fiber adsorption matrix is inversely proportional to the crystallinity of fiber bundles. Secondly, the crystallinity of microstructural fiber bundles is inversely proportional to the “free” hydroxyl groups in plant fiber adsorption materials. Thirdly, the crystallinity of the microstructural fiber bundle is positively correlated with the toughness of the plant fiber adsorbent (Fig 8). Thus, the optimal equilibrium value (or interval) may be associated with hygroscopicity, crystallinity, toughness, microfiber length and "free" hydroxyl groups. Future development of plant fiber adsorption materials should take into consideration the need for high-throughput nucleic acid extraction and detection, with particular reference to cases such as the current COVID-19 pandemic, in addition to the kits used in general scientific research laboratories. On one hand, the plant fiber adsorbents identified in this study and their extraction schemes may exhibit a certain practicability with respect to meeting the experimental needs of general scientific research laboratories. On the other hand, developing new tools such as plant fiber-based magnetic beads by wrapping plant microfibers on magnetic beads, in order to meet the demands of high-throughput extraction and detection in the face of unforeseen adverse events, such as the COVID-19 pandemic, may steer related research in an entirely new direction. The suitability of the magnetic bead method for high-throughput nucleic acid extraction and detection, in situations such as the current COVID-19 epidemic, may be attributed to the controllable retraction of magnetic nanoparticles under the action of an external magnetic field. The most common magnetic beads used for nucleic acid extraction are silicon-based coated magnetic beads. The control variable method proved that bamboo fiber and cotton fiber based nucleic acid extractions were more efficient than the existing silicon-based and magnetic bead-based ones. Therefore, nucleic acid extractions based on bamboo fiber, cotton fiber or a similar plant fiber wrapped on magnetic beads to make plant fiber magnetic beads, which are intended to extract nucleic acids without involving centrifugal steps, are more efficient than the existing magnetic bead technology-based nucleic acid extractions. Improving the efficiency of nucleic acid extraction would expectedly lead to improved accuracy of downstream biological detection.

## 4. Conclusion

The current study demonstrated the tight correlation of crystallinity and hygroscopicity in PF-BAs with their DNA extraction capacity, and proposed the concept of DNA adsorption on PF-BAs, for the first time. This paper also provides a complete picture of the DNA extracting efficiency of PF-BAs, as represented by BP and DC, and demonstrated the detailed process involved in mining new PF-BAs (i.e., BP and DC) for DNA extraction, via the specific extracting system that was developed. Our innovative PF-BA extracting system showed a DNA extracting efficiency which was 4.2 times that of the approaches being currently used. The increase in the time taken to extract DNA indicates an increase in the accuracy of achieving the targeted DNA extraction as well as in the reliability of related downstream analysis and detection. Also, the purity of DNA extracted via the new method met the suggested standards for an OD260/OD280 between 1.7 and 2.0, and an OD260/OD230 above 2.0, indicating that the new method could be applicable in a wide range of downstream experiments. This new protocol could be converted into a new DNA extraction product with less economical cost, quadruple extraction efficiency and rapid DNA extraction. Further enhancements of this new DNA extraction technique may be achieved as follows: (i) considering the very high correlation between the quantity of DNA extracted, AmorI and hygroscopicity, an “Ideal” PF-BA could be possibly manufactured; (ii) replacement of the centrifugal force (e.g., pressure) to simplify the operating machinery may help increase the sample number being processed at one time; (iii) an all-in-one detection instrument could be designed based on currently available instruments.

## Supporting information

S1 Fig The principle underlying CC (centrifuge column) and MB (magnetic bead) methods for DNA extraction. (TIF)

S2 Fig (a) The customized 7 mm hole puncher (HONGXUAN). (b). Representative contents of the assembled extraction spin column sets.
(TIF)

S3 Fig Schematic of the main technical routine. (TIF)

S4 Fig Overview of protocols for the five current DNA extraction approaches. (TIF)

S5 Fig (a) The capacities of lysis buffer C (pH 4 to 10), and elution buffer Q (pH 9) for extracting DNA on DC, using the new protocol. (b). DNA electrophoresis of the related samples (Marker, *lyC4,5,6,7,8,9,10DC; *lyC and the number refers to lysis buffer C and the pH of lysis buffer C). (c) The OD260/OD280 (black) and OD260/OD230 (purple) purity ratios of the related samples.
(TIF)

S6 Fig (a) The capacities of lysis buffer N (pH 4 to 10) and elution buffer Q (pH 9) for extracting DNA on DC, using the new protocol. (b) DNA electrophoresis of the related samples (Marker, *lyN4,5,6,7,8,9,10DC; *lyC and the number refers to lysis buffer N and the pH of lysis buffer N). (c) The OD260/OD280 (black) and OD260/OD230 (purple) purity ratios of the related samples.
(TIF)

S7 Fig (a) The capacity of lysis buffer N (pH 5–7) and elution buffer Q (pH 6–10) for extracting DNA on BP and DC, using the new protocol. (b) The OD260/OD280 (black) and OD260/OD230 (purple) purity ratios of the related samples. (c) DNA electrophoresis of the related sample from BP as the adsorbent (Marker; lyN5,6,7BPe*6; lyN5,6,7BPe7; lyN5,6.7BPe8; lyN5,6,7BPe9). (d) DNA electrophoresis of the related sample from DC as the adsorbent (Marker; lyN5,6,7DCe6; lyN5,6,7DCe7; lyN5,6.7DCe8; lyN5,6,7DCe9). *e refers to the elution buffer e.
(TIF)

S8 Fig (a) Crystallinity shown via XRD analysis. (b) Contact angle was determined using a contact angle goniometer. (c) Comprehensive histogram and line chart allowing visualization of the situation among extracted DNA, AmorI and CA in BP and DC. (d) Linear fitting between AmorI and the quantity of extracted DNA. (e) Linear fitting between CA and the quantity of extracted DNA.
(TIF)

S9 Fig The proposed principle of the adsorption of DNA onto PF-BA. (TIF)

S1 Raw images (PDF)

## References

[1] Alias AB, Chiang CE, Huang HY, Lin KT, Lu PJ, Wang YW, et al. Extraction of Cell-free Dna from An Embryo-culture Medium Using Micro-scale Bio-reagents on Ewod. Sci Rep. 2020;10(1):9708. doi: 10.1038/s41598-020-66779-z 32546702 PMC7298037

[2] Tan SC, Yiap BC. DNA, RNA, and Protein Extraction: The Past and The Present. Journal of Biomedicine and Biothechnology. 2009;2009(574398). 10.1155/2009/574398 PMC2789530 20011662

[3] Ali N, Rampazzo R. C. P., Costa A. D. T. & Krieger M. A. Current Nucleic Acid Extraction Methods and Their Implications to Point-of-Care Diagnostics. BioMed Res Int 2017;2017: 9306564. doi: 10.1155/2017/9306564 28785592 PMC5529626

[4] Nouws S, Bogaerts B, Verhaegen B, Denayer S, Pierard D, Marchal K, et al. Impact of DNA extraction on whole genome sequencing analysis for characterization and relatedness of Shiga toxin-producing Escherichia coli isolates. Sci Rep. 2020;10(1):14649. doi: 10.1038/s41598-020-71207-3 32887913 PMC7474065

[5] Fierro RG, Lopez DT, Deserio D, Liebana E, Rizzi V, Guerra B. Outcome of EC/EFSA questionnaire (2016) on use of Whole Genome Sequencing (WGS) for food-and waterborne pathogens isolated from animals, food, feed and related environmental samples in EU/EFTA countries. EFSA. 2018;EN-1432.

[6] Revez J, Espinosa L, Albiger B, Claire KL, Struelens MJ, Group ENMFPaE. Survey on the Use of Whole-Genome Sequencing for Infectious Diseases Surveillance: Rapid Expansion of European National Capacities, 2015–2016. Front Public Health. 2017;5(347). doi: 10.3389/fpubh.2017.00347 29326921 PMC5741818

[7] Nouws S, Bogaerts B, Verhaegen B, Denayer S, Crombé F, Rauw KD, et al. The Benefits of Whole Genome Sequencing forFoodborne Outbreak Investigation from thePerspective of a National Reference Laboratory in a Smaller Country. Foods. 2020;9(1030). 10.3390/foods9081030 PMC7466227 32752159

[8] Lee H, Na W, Park C, Park KH, Shin S. Centrifugation-free extraction of circulating nucleic acids using immiscible liquid under vacuum pressure. Sci Rep. 2018;8(1):5467. doi: 10.1038/s41598-018-23766-9 29615736 PMC5883035

[9] Ziegler A, Koch A, Krockenberger K, Großhennig A. Personalized medicine using DNA biomarkers: a review. Hum Genet 2012;131:1627–38. doi: 10.1007/s00439-012-1188-9 22752797 PMC3432208

[10] Sidransky D. Nucleic acid-based methods for the detection of cancer. Science. 1997;278:1054–8. doi: 10.1126/science.278.5340.1054 9353179

[11] Schwarzenbach H, Hoon DS, Pantel K. Cell-free nucleic acids as biomarkers in cancer patients. Nature Reviews Cancer. 2011;11:426–37. doi: 10.1038/nrc3066 21562580

[12] Martzy R, Bica-Schroder K, Palvolgyi AM, Kolm C, Jakwerth S, Kirschner AKT, et al. Simple lysis of bacterial cells for DNA-based diagnostics using hydrophilic ionic liquids. Sci Rep. 2019;9(1):13994. doi: 10.1038/s41598-019-50246-5 31570727 PMC6768989

[13] Melzak KA, Sherwood CS, Turner RFB, Haynes CA. Driving forces for dna adsorption to silica in perchlorate solution. Journal of colloid and interface science. 1996;181:635–44.

[14] Husain A. A novel approach to minimize the false negative COVID-19 diagnosis by inclusion of specific cell markers and multiple sample collection. MethodsX. 2021;8:101270. doi: 10.1016/j.mex.2021.101270 33614422 PMC7881297

[15] Di Paolo M, Iacovelli A, Olmati F, Menichini I, Oliva A, Carnevalini M, et al. False-negative RT-PCR in SARS-CoV-2 disease: experience from an Italian COVID-19 unit. ERJ Open Res. 2020;6(2). doi: 10.1183/23120541.00324-2020 32685435 PMC7357270

[16] Hernández EJ, Mora-Umaña F, Albertazzi F, Karkashian JP, Ramírez P. Comparative Analysis of Three Different Total Nucleic Acid Extraction Protocols for the Diagnosis of Geminiviruses in Squash (Cucurbita moschata). Journal of Phytopathology. 2012;160(1):19–25.

[17] Tsai HP, Tsai YY, Lin IT, Kuo PH, Chen TY, Chang KC, et al. Comparison of Two Commercial Automated Nucleic Acid Extraction and Integrated Quantitation Real-Time PCR Platforms for the Detection of Cytomegalovirus in Plasma. PLoS One. 2016 11(8):e0160493. doi: 10.1371/journal.pone.0160493 27494707 PMC4975419

[18] Haukanes BI, Kvam C. Application of Magnetic beads in bioassays. Bio/technology. 1993;11(1):60–3. doi: 10.1038/nbt0193-60 7763485

[19] Jue E, Witters D, Ismagilov RF. Two-phase wash to solve the ubiquitous contaminant-carryover problem in commercial nucleic-acid extraction kits. Sci Rep. 2020;10(1):1940. doi: 10.1038/s41598-020-58586-3 32029846 PMC7004994

[20] Price CW, Leslie D. C. & Landers J. P. Nucleic acid extraction techniques and application to the microchip. Lab a chip. 2009;9:2484–94. 10.1039/b907652m 19680574

[21] Lee H, Park C, Na W, Park KH, Shin S. Precision cell-free DNA extraction for liquid biopsy by integrated microfluidics. NPJ Precis Oncol. 2020;4:3. doi: 10.1038/s41698-019-0107-0 32133418 PMC7039987

[22] Devonshire AS, Whale AS, Gutteridge A, Jones G, Cowen S, Foy CA, et al. Towards standardisation of cell-free DNA measurement in plasma: controls for extraction efficiency, fragment size bias and quantification. Analytical & Bioanalytical Chemistry. 2014;406: 6499–512. doi: 10.1007/s00216-014-7835-3 24853859 PMC4182654

[23] Mauger F, Dulary C, Daviaud C, Deleuze JF, Tost J. Comprehensive evaluation of methods to isolate, quantify, and characterize circulating cell-free DNA from small volumes of plasma. Anal Bioanal Chem. 2015;407:6873–8. doi: 10.1007/s00216-015-8846-4 26123439

[24] Sherwood JL, Corcoran C, Brown H, Sharpe AD, Musilova M, Kohlmann A. Optimised pre-analytical methods improve KRAS mutation detection in circulating tumour DNA (ctDNA) from patients with non-small cell lung cancer (NSCLC). PLoS ONE. 2016;11:e0150197. doi: 10.1371/journal.pone.0150197 26918901 PMC4769175

[25] Tanaka T, Sakai R, Kobayashi R, Hatakeyama K, Tadashi M. Contributions of phosphate to dna adsorption-desorption behaviors on aminosilane-modified magnetic nanoparticles. Langmuir. 2009;25:2956–61. doi: 10.1021/la8032397 19437706

[26] Sun. Ning DC, Liu Yi, Zhao Xiali, Tang Yan, Liu Renxiao, Xia Qiang, et al. Optimization of influencing factors of nucleic acid adsorption onto silica-coated magnetic particles: application to viral nucleic acid extraction from serum. J Chromatogr A. 2014;1325:31–9. doi: 10.1016/j.chroma.2013.11.059 24360257

[27] Sun N, Deng C, Liu Y, Zhao X, Tang Y, Liu R, et al. Optimization of influencing factors of nucleic acid adsorption onto silica-coated magnetic particles: application to viral nucleic acid extraction from serum. Journal of chromatography A. 2014;1325:31–9. doi: 10.1016/j.chroma.2013.11.059 24360257

[28] He H, Li R, Chen Y, Pan P, Tong W, Dong X, et al. Integrated DNA and RNA extraction using magnetic beads from viral pathogens causing acute respiratory infections. Sci Rep. 2017;7:45199. doi: 10.1038/srep45199 28332631 PMC5362898

[29] Shan Z, Zhou Z, Chen H, Zhang Z, Zhou Y, Wen A, et al. PCR-ready human DNA extraction from urine samples using magnetic nanoparticles. Journal of Chromatography B 2012;881: 63–8. doi: 10.1016/j.jchromb.2011.11.042 22189018

[30] Green MR, Sambrook J. Isolation and Quantification of DNA. Cold Spring Harb Protoc. 2018;2018(6). doi: 10.1101/pdb.top093336 29858343

[31] Sambrook MRGaJ. Molecular Cloning: A Laboratory Manual (Four Edition). New York, USA: Cold Spring Harbor Laboratory Press; 2017.

[32] Mason MG, Botella JR. Rapid (30-second), equipment-free purification of nucleic acids using easy-to-make dipsticks. Nat Protoc. 2020;15(11):3663–77. doi: 10.1038/s41596-020-0392-7 33005038 PMC7528719

[33] Zou Y, Mason MG, Wang Y, Wee E, Turni C, Blackall PJ, et al. Nucleic acid purification from plants, animals and microbes in under 30 seconds. PLoS Biol. 2017;15(11):e2003916. doi: 10.1371/journal.pbio.2003916 29161268 PMC5697807

[34] Shi R, Lewis RS, Panthee DR. Filter paper-based spin column method for cost-efficient DNA or RNA purification. PLoS One. 2018;13(12):e0203011. doi: 10.1371/journal.pone.0203011 30532193 PMC6286138

[35] Qiu X, Mauk MG. An integrated, cellulose membrane-based PCR chamber. Microsystem Technologies. 2014;21(4):841–50.

[36] Gan W, Zhuang B, Zhang P, Han J, Li CX, Liu P. A filter paper-based microdevice for low-cost, rapid, and automated DNA extraction and amplification from diverse sample types. Lab Chip. 2014;14(19):3719–28. doi: 10.1039/c4lc00686k 25070548

[37] Warburg O, Christian W. Isolierung und Kristallisation des Garungsterments Enolase. Biochem Z 1942;310:384–421.

[38] Shen C. Detection and Analysis of Nucleic Acids. Diagnostic Molecular Biology 2019. p. 167–85.

[39] Glasel JA. Validity of nucleic acid puritites monitored by 260nm/280nm absorbance ratios. Biotechniques. 1995;18(1):62–3. 7702855

[40] Fu B, Wang J, Duan J, Wang C, Chen M, Tang Z, et al. Technical specification for quality evaluation of nucleic acid extraction and purification. China: State Administration for Market Regulation, Standardization Administration of China; 2019.

[41] Meng Q, Fu B, Hu S, Ge X, Tan X, Niu C, et al. General rules for evaluation of nucleic acid extraction and purification methods. China: State Administration for Market Regulation, Standardization Administration of China; 2019.

[42] Lysis buffer for genomic DNA. Cold Spring Harbor Protocols.2010.

[43] QIAGEN. What is the composition of buffer AE?: QIAGEN; 2022 [The composition of Buffer AE]. https://www.qiagen.com/kr/resources/faq?id=c484a4ad-6f46-4cb5-96f3-875b72f41512&lang=en.

[44] Sluiter A, Hames B, Ruiz R, Scarlata C, Sluiter J, Templeton D, et al. Determination of Structural Carbohydrates and Lignin in Biomass NREL. 2012;TP-510-42618.

[45] Zhang W, He J, Jiang H, Xiong P. Determination of cellulose and hemicellulose content in wood fiber substance. Jiangsu Agricultural Sciences. 2017(5):281–4.

[46] Agricultural biomass raw materials- Determination of cellulose, hemicellulose, and lignin. Ministry of agriculture and rural affairs of the People’s Republic of China; 2019.

[47] Zhang S, Qi H, Liu Y, Cheng W. Impact on timber crystallization performance of superheated steam-treated wood under high temperature and pressure. Journal of Nanjing Forestry University (Natural Science Edition). 2010(5).

[48] Chand N, Fahim M. Tribology of Natural Fiber Polymer Composites. Second ed: Woodhead Publishing; 2020.

[49] Ren H, Ren M, Li Z, Ning J, Zhang Y. Sphalerite/silica interaction: Zeta potential distribution measurement. Rare Metals. 2014;35(9):729–34.

[50] Suganthi KS, Rajan KS. Temperature induced changes in ZnO–water nanofluid: Zeta potential, size distribution and viscosity profiles. International Journal of Heat and Mass Transfer. 2012;55(25–26):7969–80.

[51] Alberts B, Bray D, Hopkin K, Johnson A, Lewis J, Reff M, et al. Essential Cell Biology. Third ed: Garland Science; 2010.

[52] Tan SC, Ong CE, Hay YK, Yiap BC. Cellulose And Its Application In Biomolecules Purification. International Research Journal of Applied and Basic Sciences. 2013;7(5):267–76.

[53] Holtzapple MT. Cellulose. In: Caballero B, editor. Encyclopedia of Food Sciences and Nutrition, Second Edition 2003. p. 998–1008.

[54] Chang R, Overby J. General Chemistry. Sixth ed: McGraw-Hill; 2011. p. 320–56.

