# Supplementary figures and images for: Improved DNA extraction on bamboo paper and cotton is tightly correlated with their crystallinity and hygroscopicity
Source: PLoS One. 2022 Nov 7;17(11):e0277138. doi: 10.1371/journal.pone.0277138 (PMC9639815; doi:10.1371/journal.pone.0277138)

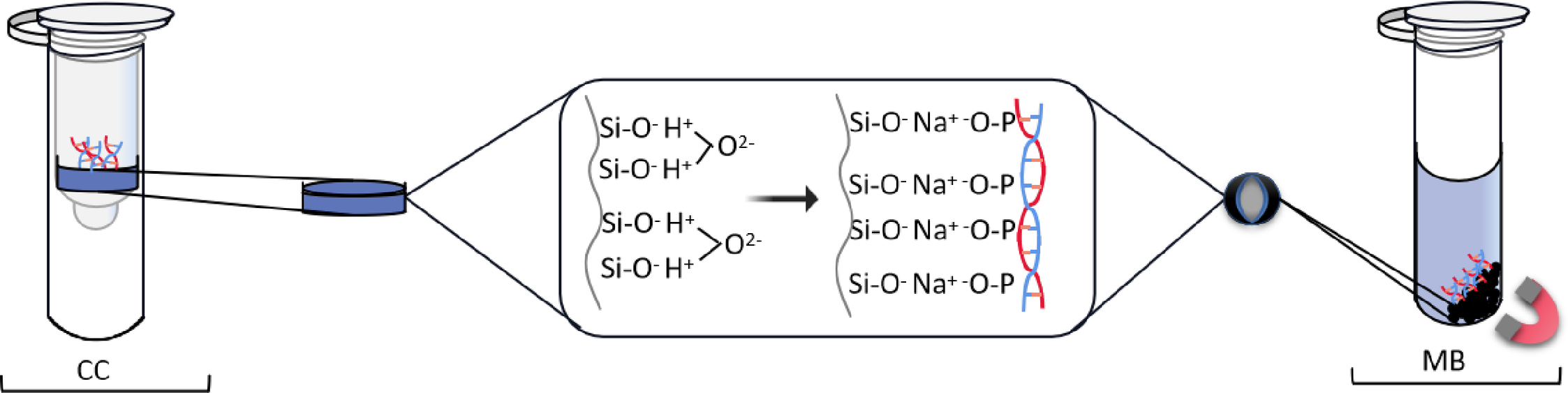

Supplement: S1 Fig — (TIF) [file pone.0277138.s001.tif]

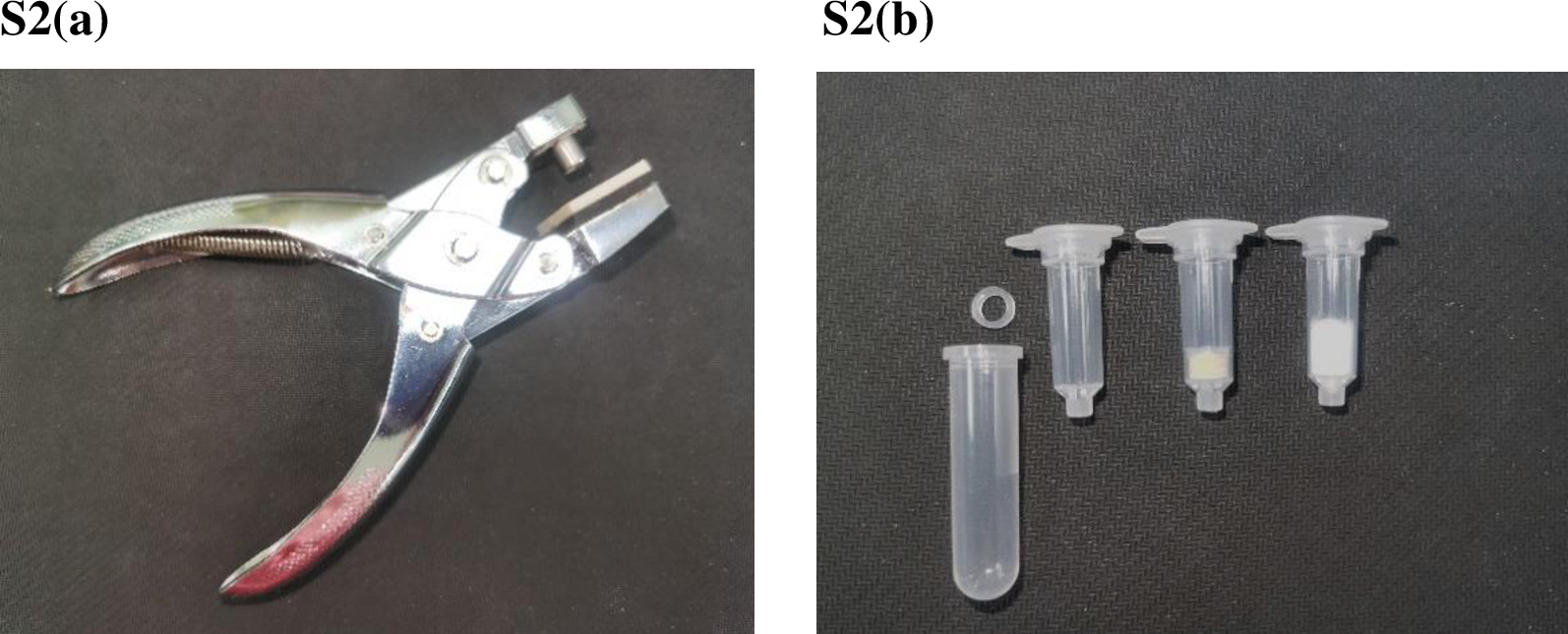

Supplement: S2 Fig — (a) The customized 7 mm hole puncher (HONGXUAN). (b). Representative contents of the assembled extraction spin column sets. (TIF) [file pone.0277138.s002.tif]

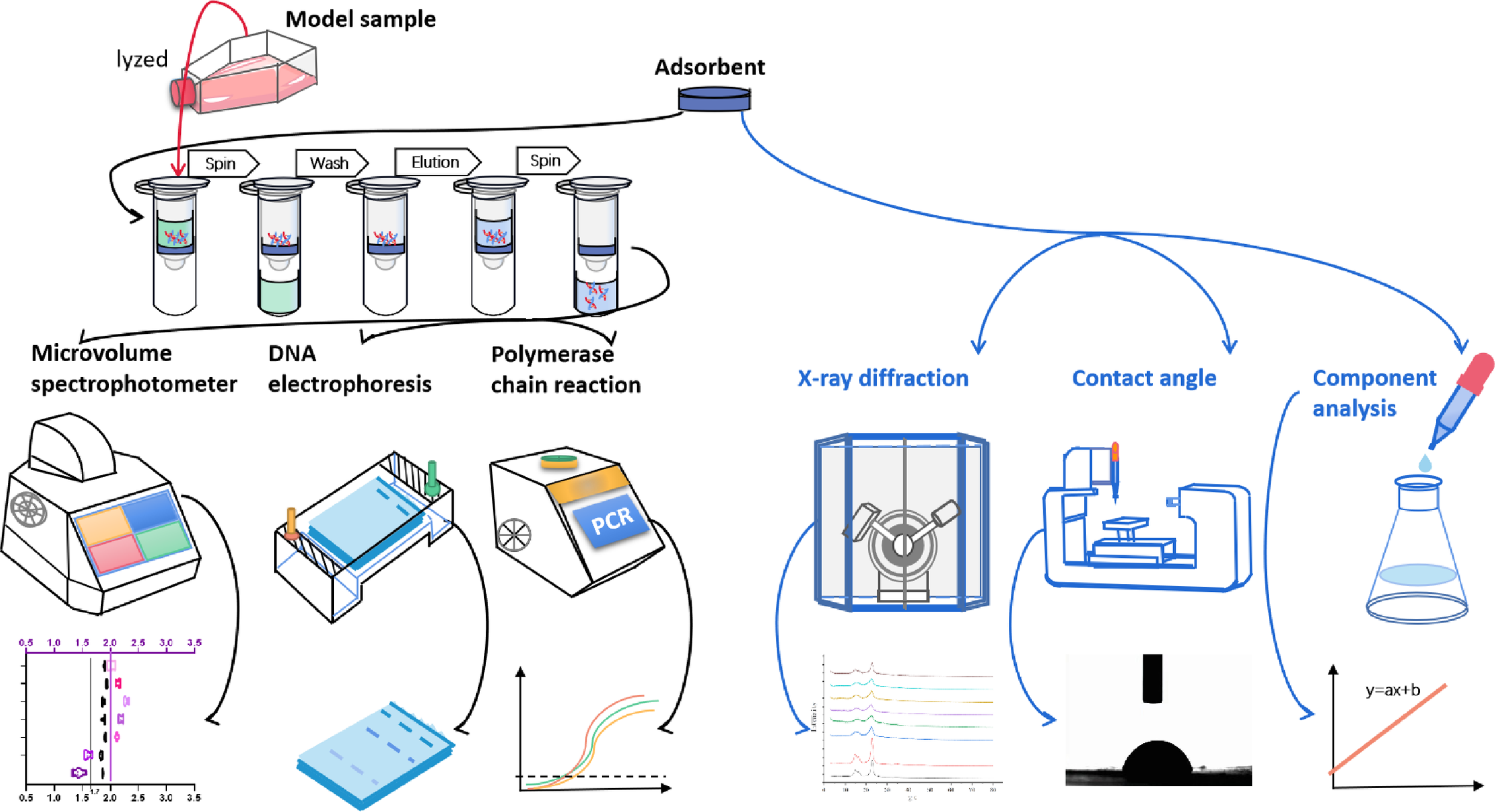

Supplement: S3 Fig — (TIF) [file pone.0277138.s003.tif]

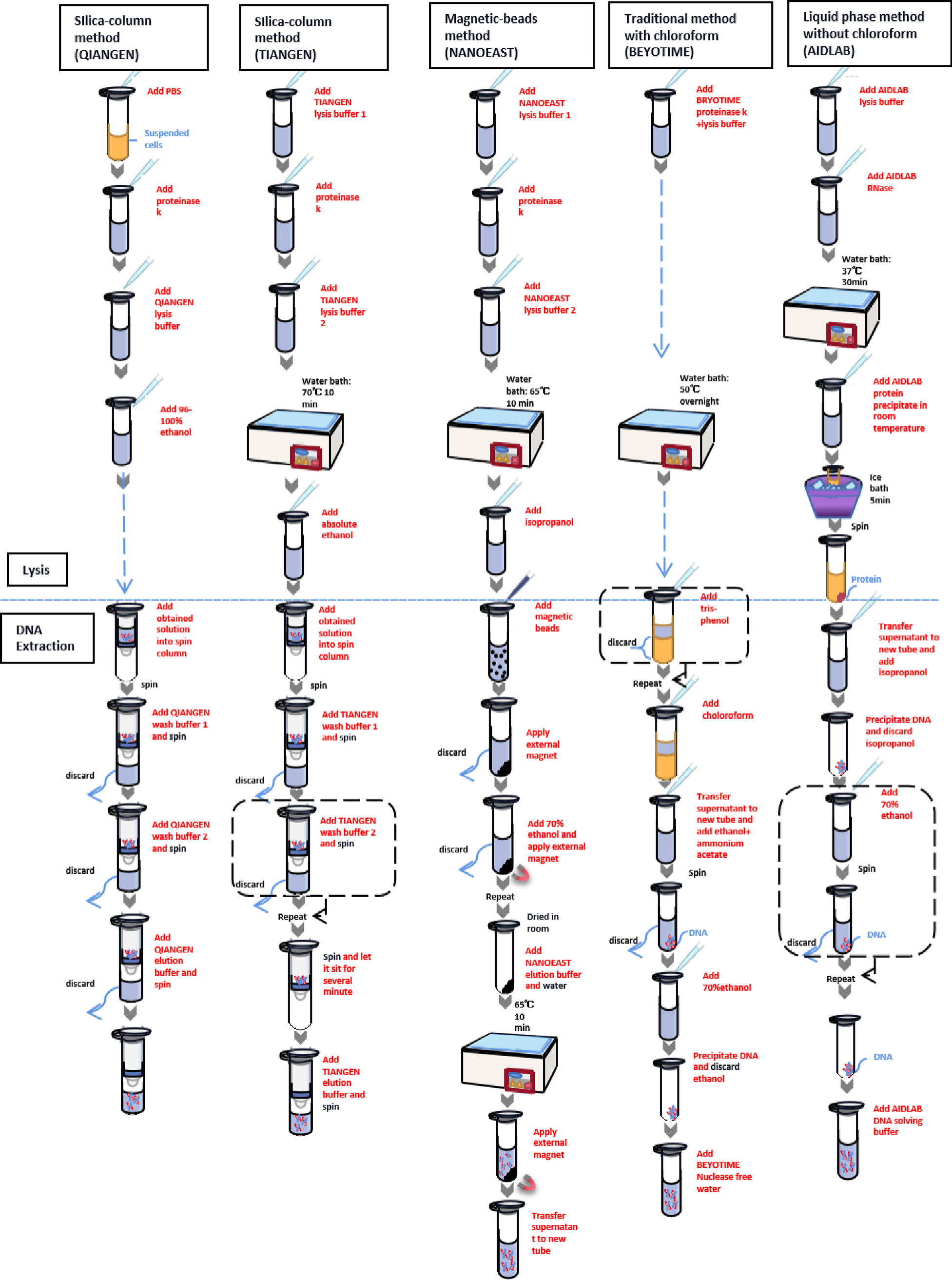

Supplement: S4 Fig — (TIF) [file pone.0277138.s004.tif]

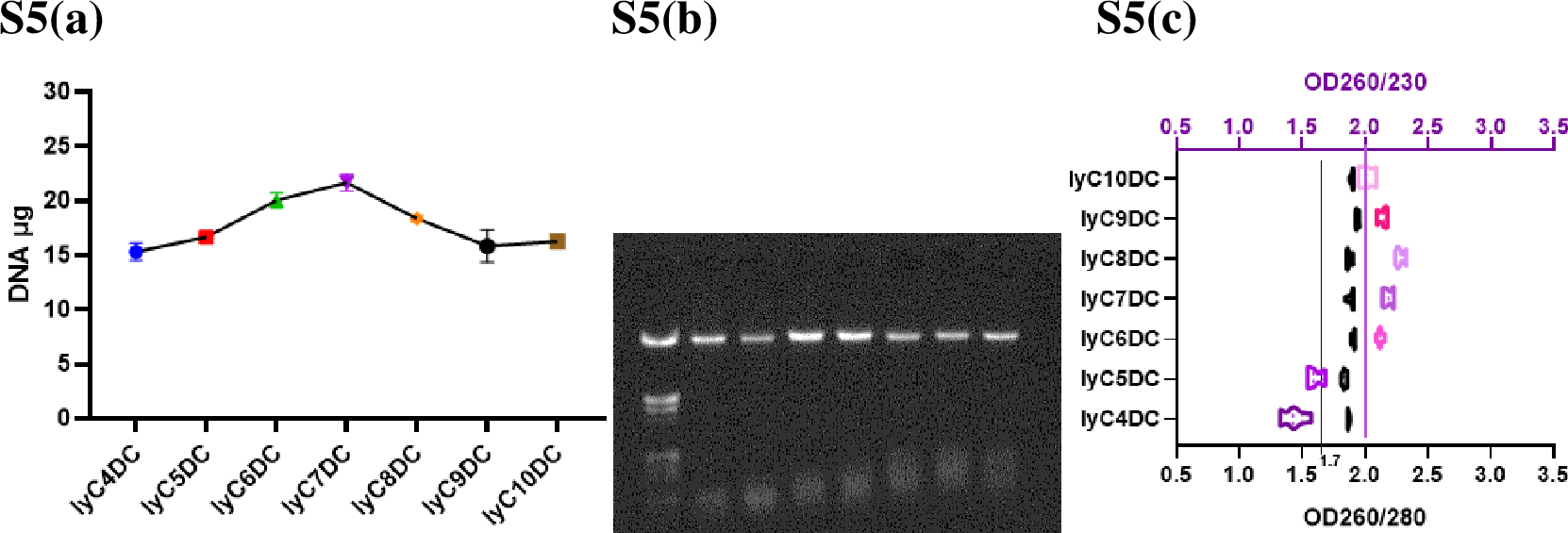

Supplement: S5 Fig — (a) The capacities of lysis buffer C (pH 4 to 10), and elution buffer Q (pH 9) for extracting DNA on DC, using the new protocol. (b). DNA electrophoresis of the related samples (Marker, *lyC4,5,6,7,8,9,10DC; *lyC and the number refers to lysis buffer C and the pH of lysis buffer C). (c) The OD260/OD280 (black) and OD260/OD230 (purple) purity ratios of the related samples. (TIF) [file pone.0277138.s005.tif]

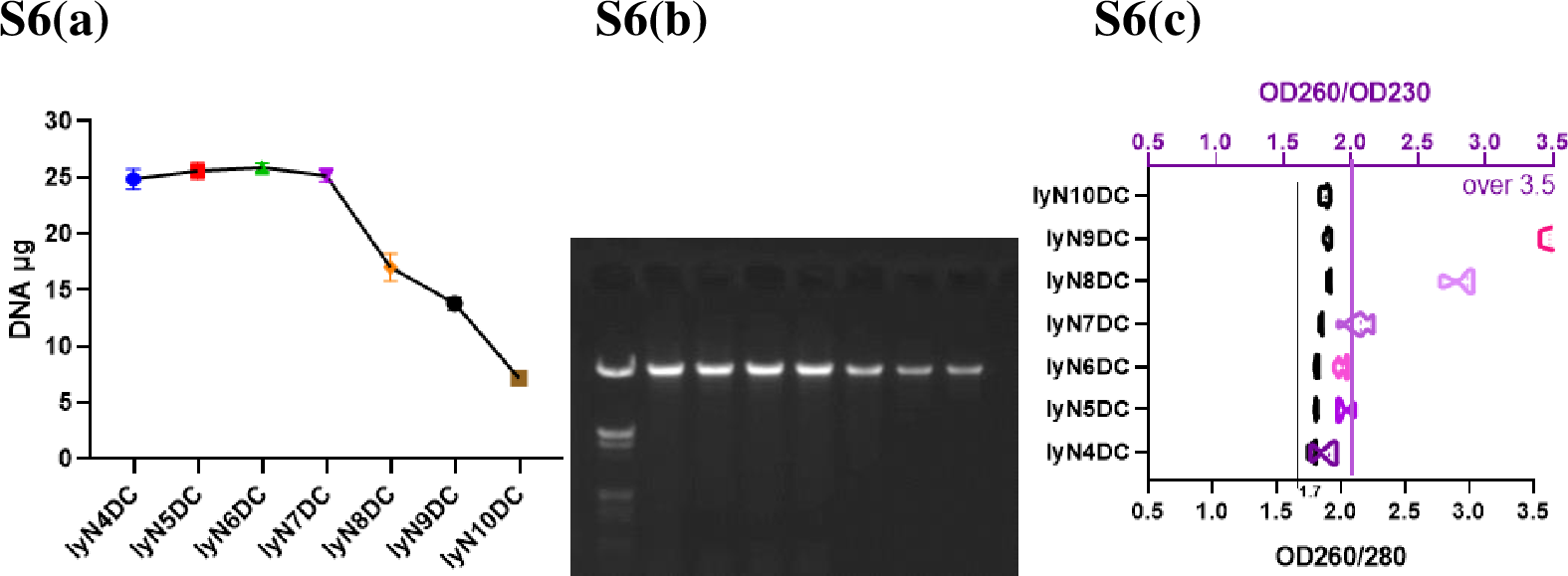

Supplement: S6 Fig — (a) The capacities of lysis buffer N (pH 4 to 10) and elution buffer Q (pH 9) for extracting DNA on DC, using the new protocol. (b) DNA electrophoresis of the related samples (Marker, *lyN4,5,6,7,8,9,10DC; *lyC and the number refers to lysis buffer N and the pH of lysis buffer N). (c) The OD260/OD280 (black) and OD260/OD230 (purple) purity ratios of the related samples. (TIF) [file pone.0277138.s006.tif]

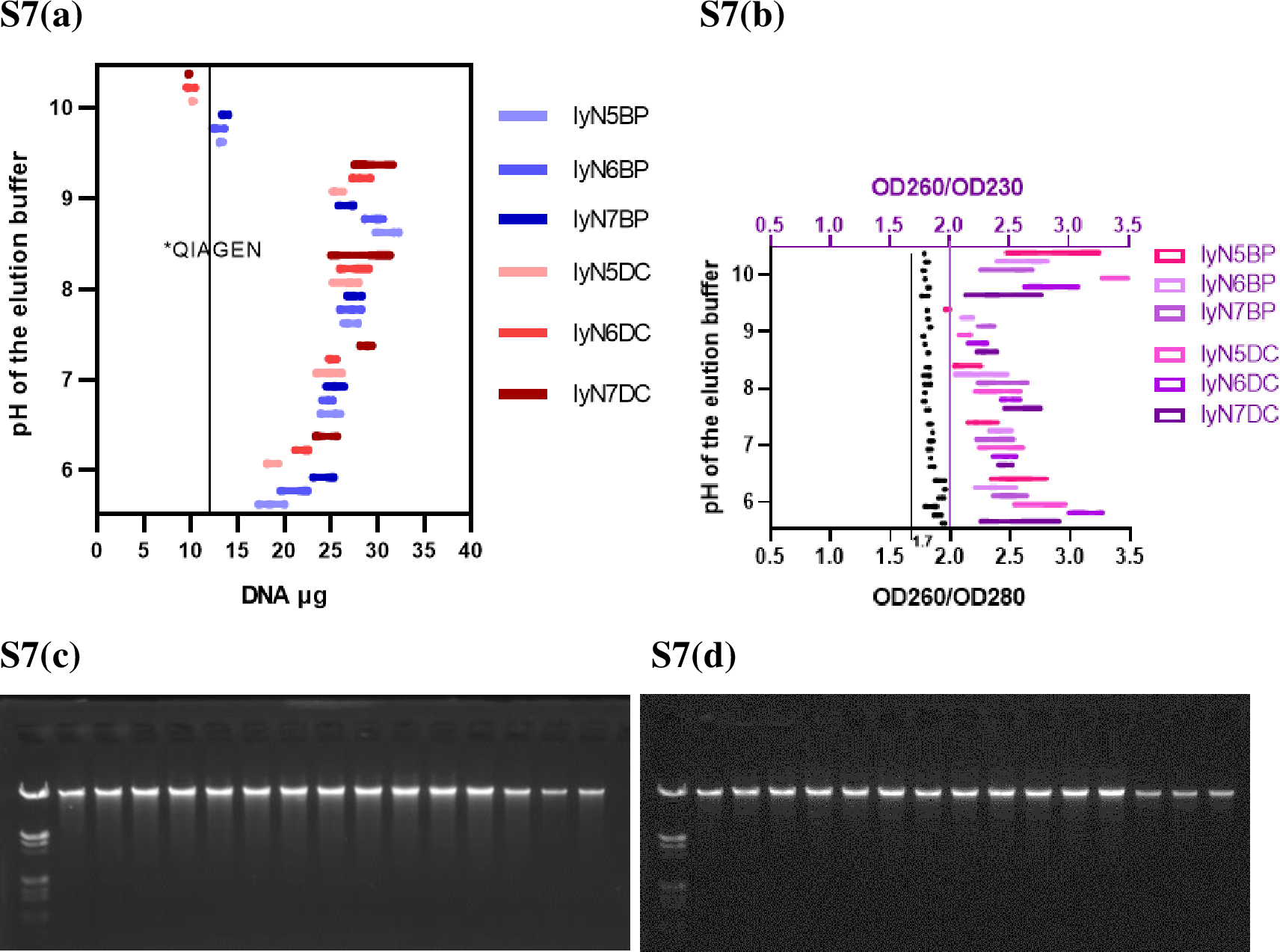

Supplement: S7 Fig — (a) The capacity of lysis buffer N (pH 5–7) and elution buffer Q (pH 6–10) for extracting DNA on BP and DC, using the new protocol. (b) The OD260/OD280 (black) and OD260/OD230 (purple) purity ratios of the related samples. (c) DNA electrophoresis of the related sample from BP as the adsorbent (Marker; lyN5,6,7BPe*6; lyN5,6,7BPe7; lyN5,6.7BPe8; lyN5,6,7BPe9). (d) DNA electrophoresis of the related sample from DC as the adsorbent (Marker; lyN5,6,7DCe6; lyN5,6,7DCe7; lyN5,6.7DCe8; lyN5,6,7DCe9). *e refers to the elution buffer e. (TIF) [file pone.0277138.s007.tif]

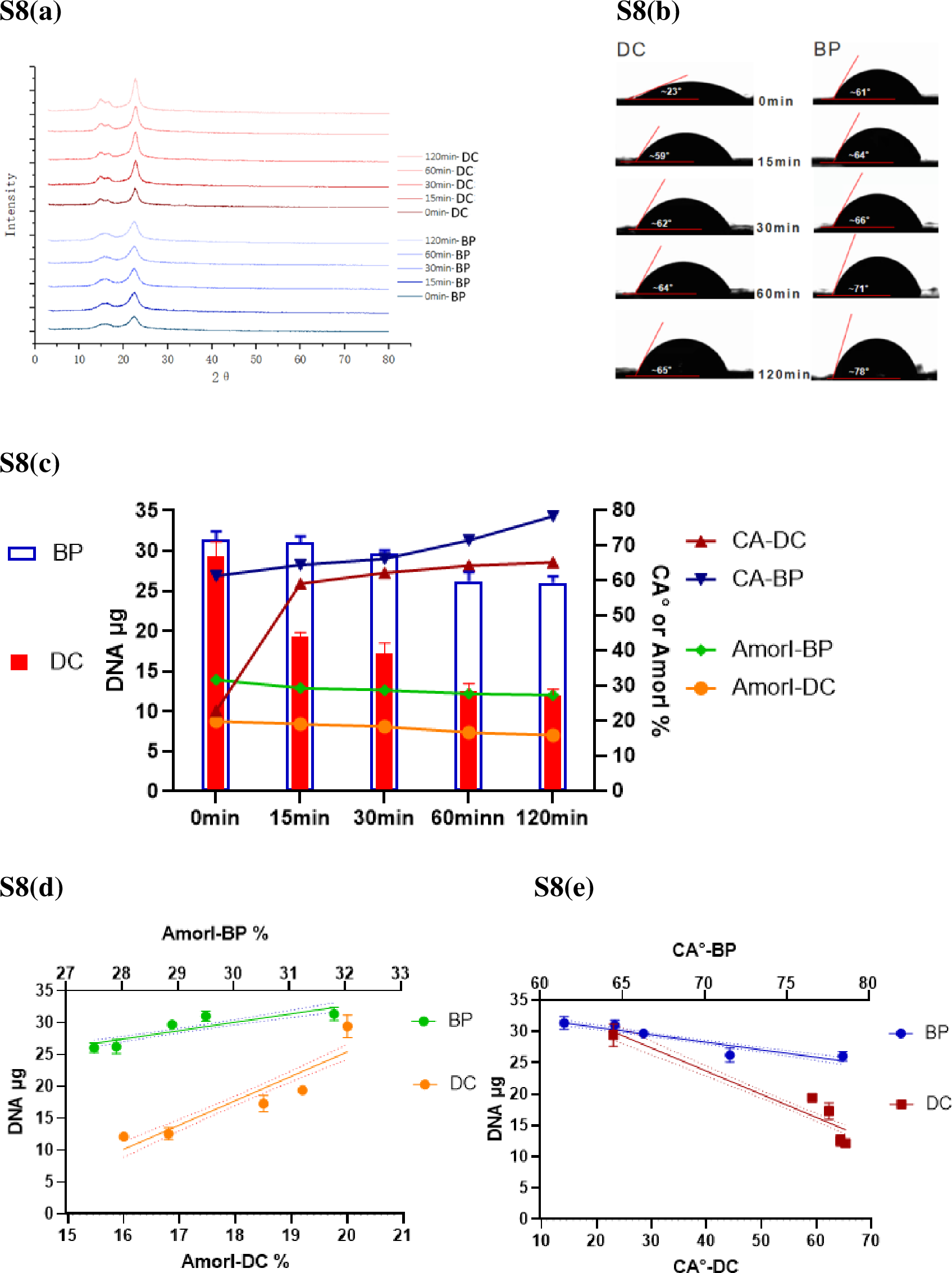

Supplement: S8 Fig — (a) Crystallinity shown via XRD analysis. (b) Contact angle was determined using a contact angle goniometer. (c) Comprehensive histogram and line chart allowing visualization of the situation among extracted DNA, AmorI and CA in BP and DC. (d) Linear fitting between AmorI and the quantity of extracted DNA. (e) Linear fitting between CA and the quantity of extracted DNA. (TIF) [file pone.0277138.s008.tif]

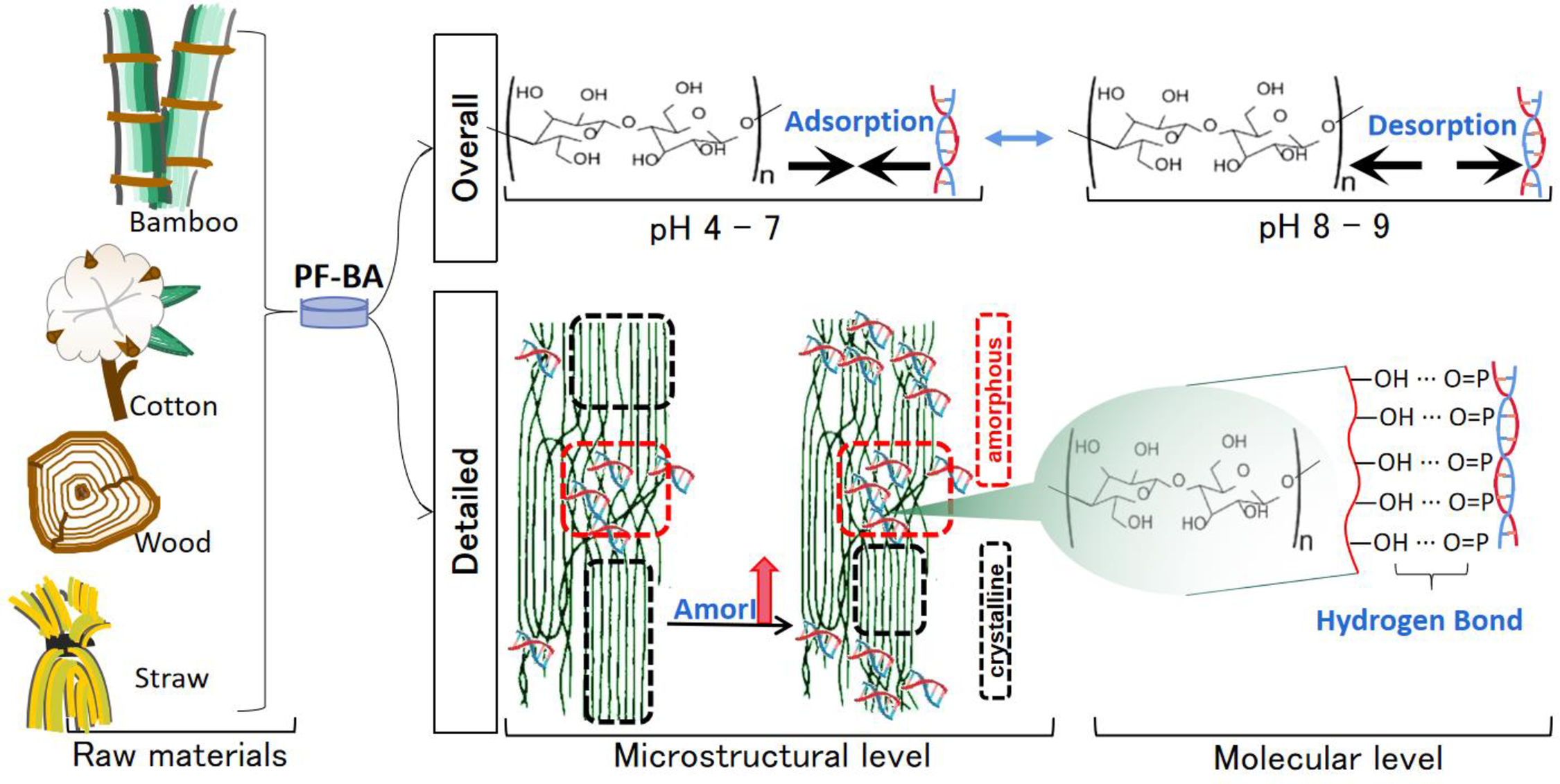

Supplement: S9 Fig — (TIF) [file pone.0277138.s009.tif]
