# Supplementary material for: Improved DNA extraction on bamboo paper and cotton is tightly correlated with their crystallinity and hygroscopicity
Source: PLoS One. 2022 Nov 7;17(11):e0277138. doi: 10.1371/journal.pone.0277138 (PMC9639815; doi:10.1371/journal.pone.0277138)
Supplement: S1 Raw images — (PDF) [file pone.0277138.s010.pdf]

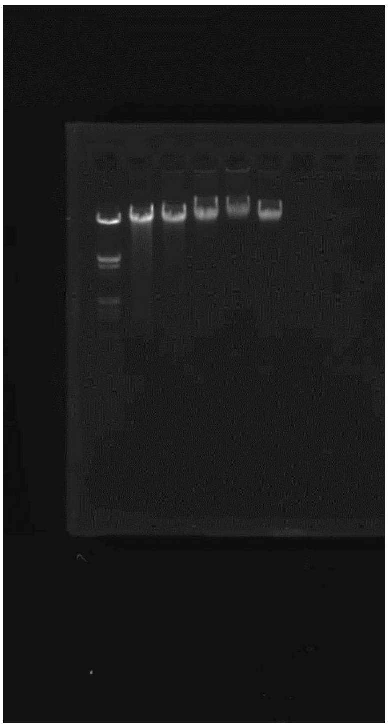

Fig. 1(b) DNA electrophoresis of the related samples (Marker, QIAGEN, TIANGEN, NANOEAST, AIDLAB, BEYOTIME). From top to bottom, each molecular weight of the marker is: 21226, 5148, 4268, 2027 and 1584 bp.

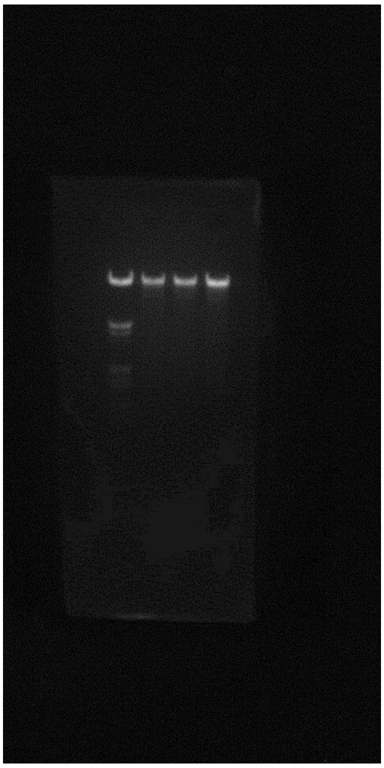

Fig. 2(b) DNA electrophoresis of the related samples (Marker,TIANGEN, NANOEAST and QIAGEN). From top to bottom, each molecular weight of the marker is: 21226, 5148, 4268, 2027 and 1584 bp.

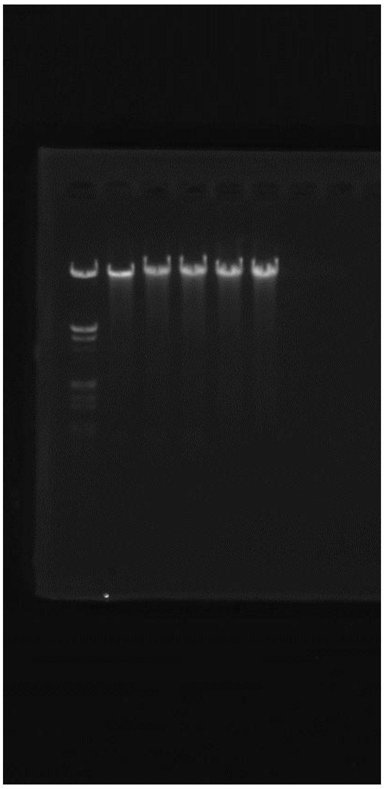

Fig. 3(b) DNA electrophoresis of related samples (Marker, *T-Grade1-p1, -p5, -p10, -p15, -p20). From top to bottom, each molecular weight of the marker is: 21226, 5148, 4268, 2027 and 1584 bp.

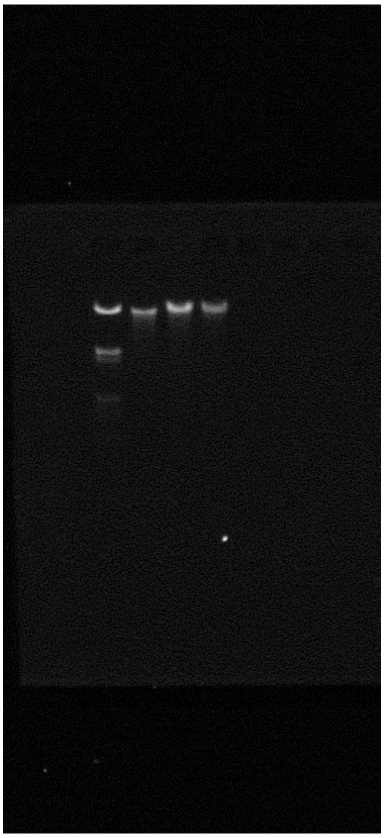

Fig. 3(d) DNA electrophoresis of related samples (Marker, T-Grade3-p1, -p5, -p10). From top to bottom, each molecular weight of the marker is: 21226, 5148, 4268, 2027 and 1584 bp.

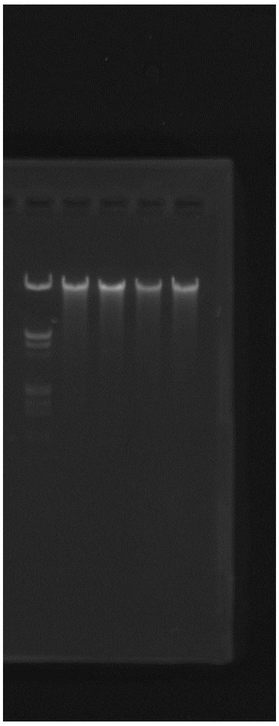

Fig. 3(f) DNA electrophoresis of related samples (Marker, T-GF/F-p1, -p5, -p10, -p15). From top to bottom, each molecular weight of the marker is: 21226, 5148, 4268, 2027 and 1584 bp.

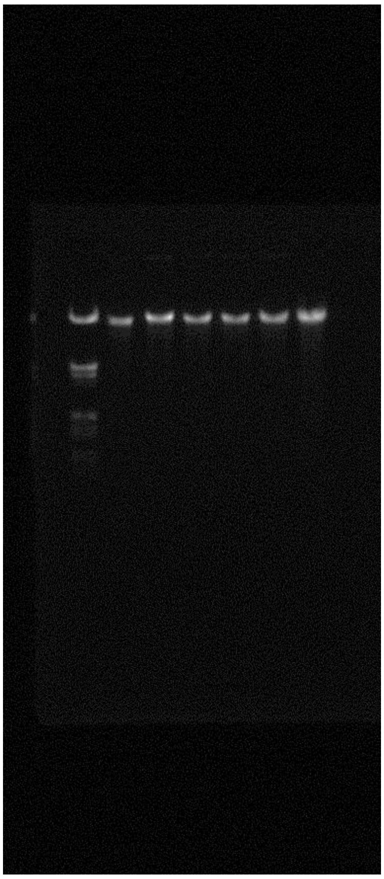

Fig. 3(h) DNA electrophoresis of related samples (Marker, T-BP-p1, -p5, -p10, -p15, -p20, -p30). From top to bottom, each molecular weight of the marker is: 21226, 5148, 4268, 2027 and 1584 bp.

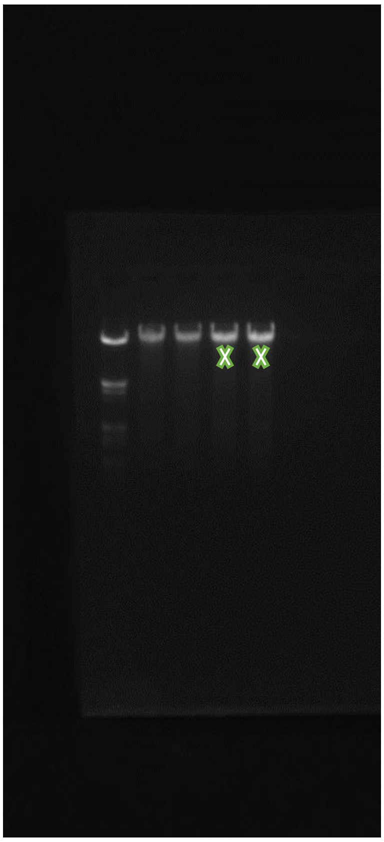

Fig. 3(j) DNA electrophoresis of related samples (Marker, WP, SP). From top to bottom, each molecular weight of the marker is: 21226, 5148, 4268, 2027 and 1584 bp.

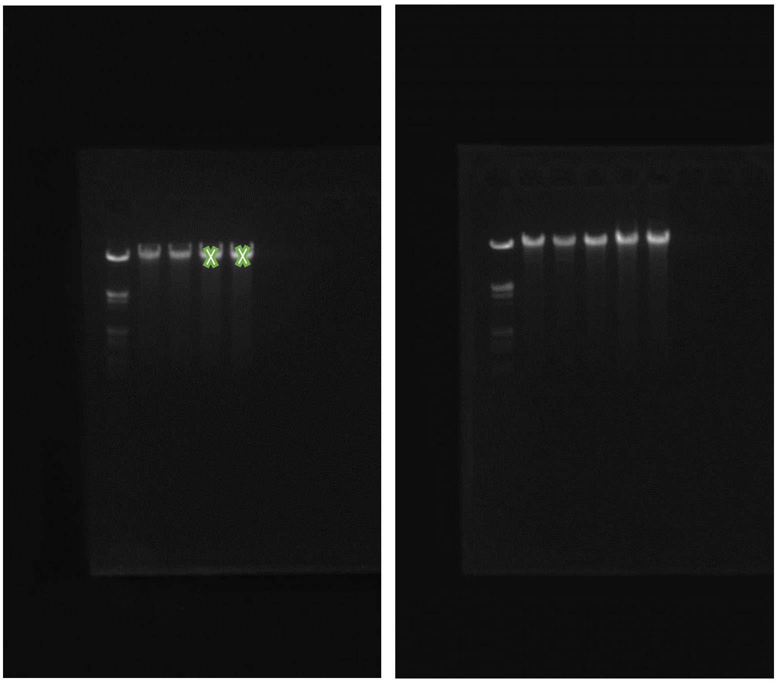

Fig. 4(b) DNA electrophoresis of related samples (Marker, *T-DC, T-BP, marker, QIAGEN, Q-NANOEAST, Q-TIANGEN, Q-DC & Q-BP). *T-, Q-, -T and -N refer to TIANGEN protocol, QIAGEN protocol, TIANGEN adsorbent and NANOEAST beads, respectively. From top to bottom, each molecular weight of the marker is: 21226, 5148, 4268, 2027 and 1584 bp.

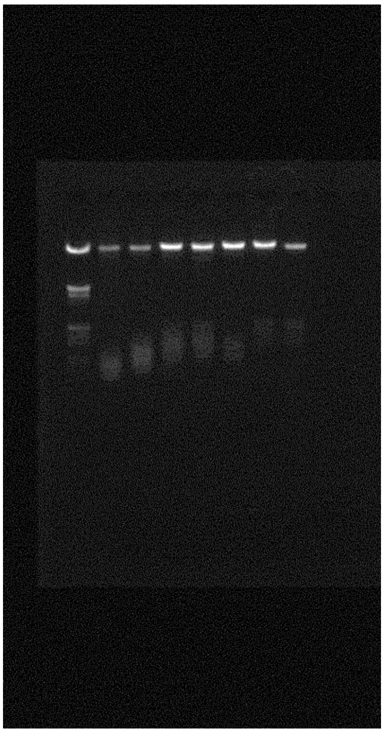

Fig. 6(b) DNA electrophoresis of the related samples (Marker, *lyC4,5,6,7,8,9,10BP; *lyC and the number refers to lysis buffer C and the pH of lysis buffer C). From top to bottom, each molecular weight of the marker is: 21226, 5148, 4268, 2027 and 1584 bp.

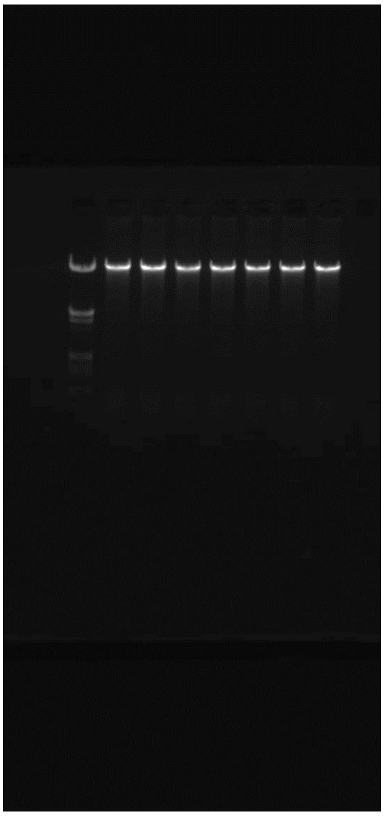

Fig. 6(e) DNA electrophoresis of the related samples (Marker, *lyN4,5,6,7,8,9,10BP; *lyN and the number refers to lysis buffer N and the pH of lysis buffer N). From top to bottom, each molecular weight of the marker is: 21226, 5148, 4268, 2027 and 1584 bp.

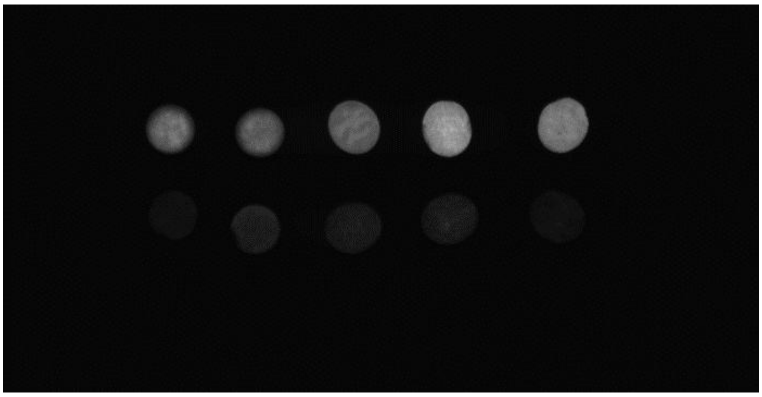

Fig. 7(b) A UV imager was used to compare DNA absorbed utilizing a single piece of PF-BA against blank controls (from left to right: G1, G3, WP, BP and SP; the corresponding blanks were placed under each sample).

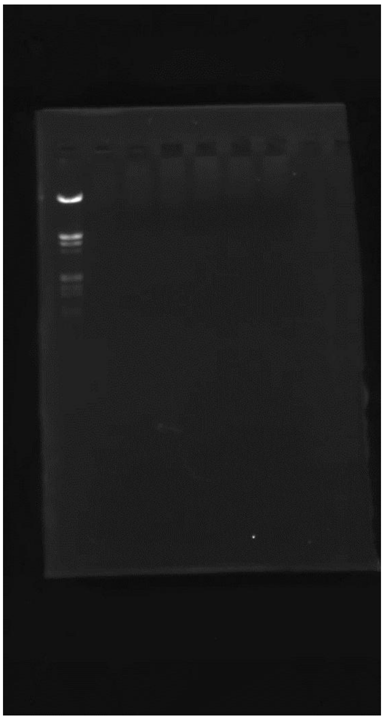

Fig. 7(c) DNA electrophoresis of the blanks of BP/DC with the new protocol (Marker; BP<n=3>; DC<n=3>). From top to bottom, each molecular weight of the marker is: 21226, 5148, 4268, 2027 and 1584 bp.

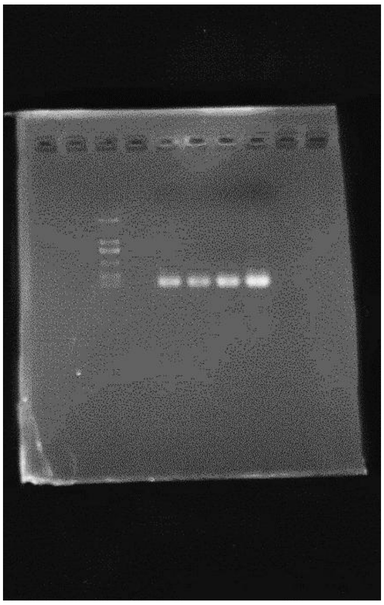

Fig. 7(d) The PCR products of negative control, TIANGEN, NANOEAST, QIAGEN and DC. From top to bottom, each molecular weight of the marker is: 2000, 1000, 750, 500, 250 and 100 bp.

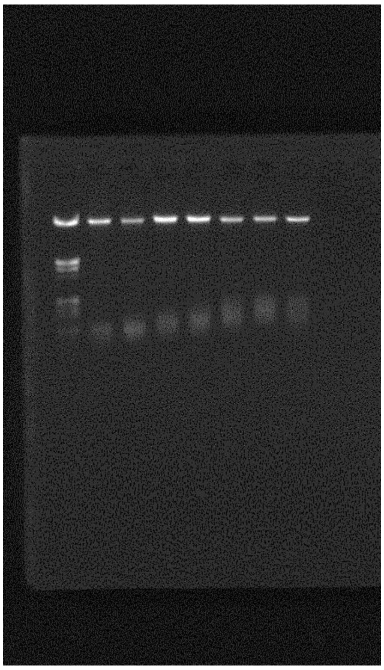

Supplement Fig. S5(b) DNA electrophoresis of the related samples (Marker, *lyC4,5,6,7,8,9,10DC; *lyC and the number refers to lysis buffer C and the pH of lysis buffer C). From top to bottom, each molecular weight of the marker is: 21226, 5148, 4268, 2027 and 1584 bp.

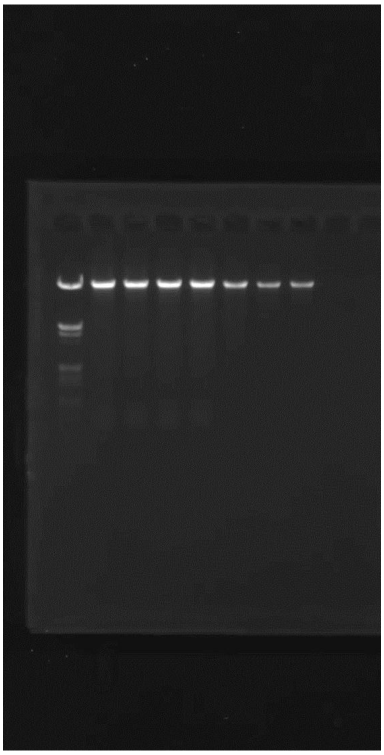

Supplement Fig. S6(b) DNA electrophoresis of the related samples (Marker, *lyN4,5,6,7,8,9,10DC; *lyC and the number refers to lysis buffer N and the pH of lysis buffer N). From top to bottom, each molecular weight of the marker is: 21226, 5148, 4268, 2027 and 1584 bp.

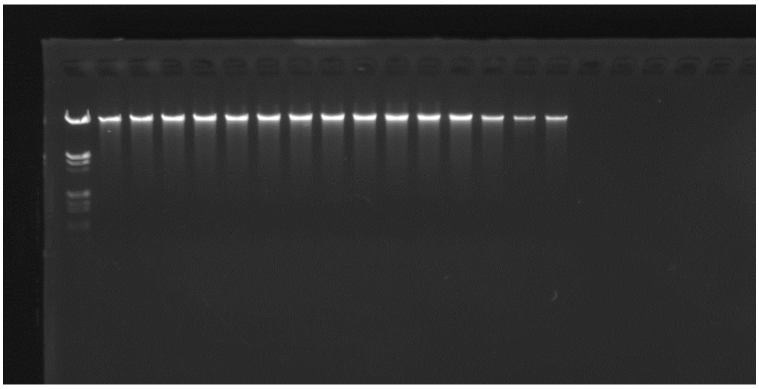

Supplement Fig. S7(c) DNA electrophoresis of the related sample from BP as the adsorbent (Marker; lyN5,6,7BPe*6; lyN5,6,7BPe7; lyN5,6.7BPe8; lyN5,6,7BPe9). *e refers to the elution buffer e. From top to bottom, each molecular weight of the marker is: 21226, 5148, 4268, 2027 and 1584 bp.

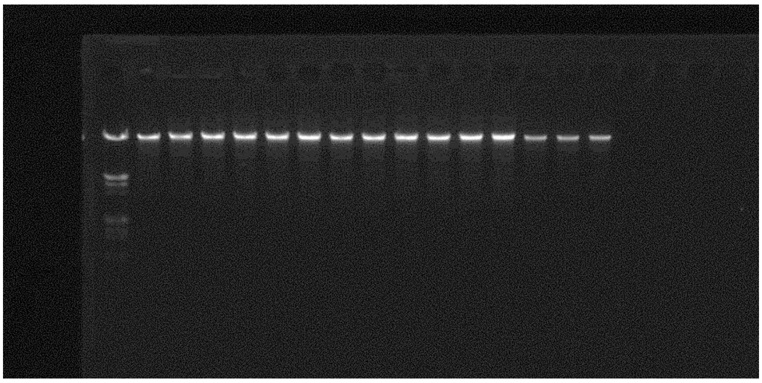

Supplement Fig. S7(d) DNA electrophoresis of the related sample from DC as the adsorbent (Marker; lyN5,6,7DCe*6; lyN5,6,7DCe7; lyN5,6.7DCe8; lyN5,6,7DCe9). *e refers to the elution buffer e. From top to bottom, each molecular weight of the marker is: 21226, 5148, 4268, 2027 and 1584 bp.
